# A kinetic and mechanistic study into the transformation of calcium sulfate hemihydrate to dihydrate

**DOI:** 10.1107/S1600577519001929

**Published:** 2019-04-05

**Authors:** Sebastian J. Gurgul, Gabriel Seng, Gareth R. Williams

**Affiliations:** a UCL School of Pharmacy, University College London, 29–39 Brunswick Square, London WC1N 1AX, UK; b Etex Group, 500 Rue Marcel Demonque, 84000 Avignon, France

**Keywords:** gypsum, calcium sulfate, hemihydrate, hydration, conversion

## Abstract

The conversion of CaSO_4_·0.5H_2_O to CaSO_4_·2H_2_O is of great importance industrially, being the reaction behind plasterboard production and the setting of medical plasters. A detailed kinetic and mechanistic study of this process was conducted using time-resolved synchrotron X-ray diffraction in this work. The CaSO_4_·2H_2_O product is very similar regardless of whether the α- or β-form of CaSO_4_·0.5H_2_O is used as the starting material, but the reaction process is very different. The induction time is usually shorter for α-CaSO_4_·0.5H_2_O than β-CaSO_4_·0.5H_2_O, and a greater conversion percentage is observed with the former (although in neither case does the reaction proceed to 100% completion). The temperature of the system, widely used in industry as an indirect measure of the extent of the hydration process, is found to be a poor pr­oxy for this, with the maximum temperature reached well before the reaction is complete. The Avrami–Erofe’ev and Gualtieri models could both be fitted to the experimental data, with the fits being substantially closer in the case of α-CaSO_4_·0.5H_2_O. The rate of reaction in the Avrami model tends to increase with the amount of gypsum seeds added to accelerate the process, and the importance of nucleation declines. The Gualtieri analysis suggested that the rate of nucleation increases substantially with the amount of seeds added, while there are less distinct changes in the rate of crystal growth. At low seed concentrations (<0.5% *w*/*w*) the rate of crystal growth is greater than the rate of nucleation, but at concentrations above 0.5% *w*/*w* nucleation is faster. These findings represent the first synchrotron study of the conversion of CaSO_4_·0.5H_2_O to CaSO_4_·2H_2_O, and will be of importance to gypsum producers globally.

## Abstract 2

A detailed kinetic and mechanistic study of the hydration of CaSO_4_·0.5H_2_O to CaSO_4_·2H_2_O is reported. This is a one-step process with no crystalline intermediate phases, and the addition of increasing amounts of CaSO_4_·2H_2_O reduces the importance of nucleation in controlling the reaction rate.

## 1. Introduction   

Together with its hydrates, calcium sulfate is a very important mineral industrially, having a broad range of applications in fields as diverse as construction, medicine, cosmetics and ceramics (Tritschler *et al.*, 2015 ▸). The CaSO_4_–H_2_O system has five crystalline phases (Van Driessche *et al.*, 2017 ▸). Four exist at room temperature: calcium sulfate dihydrate, calcium sulfate hemihydrate, γ-anhydrite and β-anhydrite. The fifth phase, α-anhydrite, is only stable above 1180°C (Wirsching, 2000 ▸).

CaSO_4_·2H_2_O (gypsum) is the basis for the plasters used in both construction and medical applications. It is prepared commercially from the hydration of Ca sulfate hemihydrate (also known as stucco). The latter has been found to exist in two forms – α and β-CaSO_4_·0.5H_2_O. α- and β-Hemihydrate are themselves prepared from the dehydration of gypsum: the α-form can be prepared under hydro­thermal conditions involving high pressure (up to 8 bar) and temperatures (120–160°C), while the β-form is generated via dry calcining at 120–180°C (Lewry & Williamson, 1994 ▸). α- and β-CaSO_4_·0.5H_2_O are reported to be structurally identical, but to have different crystal habits (Clifton, 1972 ▸). They are indistinguishable by common analytical techniques such as X-ray diffraction, but have different densities and can be distinguished through thermal analysis (Clifton, 1972 ▸). The water demand to convert them to the dihydrate form also differs (Powell & Way, 1962 ▸). α-Hemihydrate is difficult and expensive to generate and is employed to generate high-strength gypsum plasters, premium products used where strength is of prime importance: for instance for sanitaryware casing, block mould manufacture, architectural decoration, murals and sculptures, and dental plasters (Lewis *et al.*, 2006 ▸; López-Delgado *et al.*, 2014 ▸). β-Stucco is much easier and less expensive to prepare, and hence is employed in preference to the α analogue where possible. β-Hemihydrate yields plasters for plasterboard production, pottery and ceramics (Sharpe & Cork, 2006 ▸), and is produced on a significantly larger scale than α-CaSO_4_·0.5H_2_O.

Despite the great industrial importance of gypsum and its formation through the hydration of hemihydrates, remarkably little is known about this process. Further, much of what is known has been inferred through quenching reactions or indirect methods (such as the Gillmore needle apparatus or rheology) (Winkler *et al.*, 1998 ▸; Abuasi *et al.*, 1993 ▸). For instance, in industry the monitoring of the hydration reaction is evaluated using rheological properties or temperature as pr­oxy measures (Pan *et al.*, 2012 ▸). Knowing the point at which hydration is complete (the ‘setting time’) is of crucial importance to plasterboard manufacturers because it determines the length of the manufacturing line required. Any residual water left at the end of the line must be removed by a heat treatment, which is both expensive and time consuming (Wirsching, 2000 ▸). To speed up the conversion from hemihydrate to dihydrate, seed crystals of gypsum are generally added to a mixture of stucco and water; these are thought to provide extra nucleation sites, and have the additional benefit of improving the hardness of the plaster (Amathieu & Boistelle, 1986 ▸).

In general, the literature reports that the hydration process involves a dissolution/reprecipitation mechanism, with the dissolution of CaSO_4_·0.5H_2_O being relatively rapid and the subsequent precipitation of CaSO_4_·2H_2_O slower (Roch Isern & Messing, 2016 ▸). A number of authors have carried out studies seeking to understand the kinetics and mechanism of the process, but there remains significant doubt here. Hand explored a range of kinetic models, but was unable to reach any firm conclusions as to the reaction mechanism (Hand, 1994 ▸). Other authors attempting to address this question have also reached uncertain conclusions (Ridge & Surkevicius, 2007 ▸; Fujii & Kondo, 1986 ▸). For instance, Taplin (Taplin, 1965 ▸) used a model in which the particles are spherical and uniform in size and equations delivered by Polak (Polak, 1960 ▸) and Schiller (Schiller, 1964 ▸), but could not unequivocally determine the reaction mechanism, likely in part because generally gypsum seeds have a needle-like morphology. More recent insights have been obtained through quenching and cryo-transmission electron microscopy (TEM) studies. These revealed that the formation of gypsum particles begins with nanoscale amorphous clusters, which then grow into amorphous nanoparticles. Crystalline particles are subsequently formed within these amorphous particles (Saha *et al.*, 2012 ▸).

Unfortunately, it is widely known that neither quenching nor indirect methods are able to provide reliable information on the progression of chemical reactions – for instance, quenching can affect a reaction product. Studies using direct, non-invasive, probes to monitor CaSO_4_·0.5H_2_O hydration have until recently been completely lacking. However, in recent years researchers have started to explore in more detail the reaction mechanism and kinetics. One approach taken is to use nuclear magnetic resonance (NMR) to measure the *T*_1_ and *T*_2_ relaxation times of water protons during the hydration process (Saha *et al.*, 2012 ▸; Song *et al.*, 2010 ▸). These experiments revealed that α-hemihydrate plasters have fine pore structure, and the average pore size decreases during hydration, whereas the pore size in β-hemihydrate is larger (Song *et al.*, 2009 ▸). The induction period for hydration was found to be significantly longer for β-hemihydrate than for α-hemihydrate (Song *et al.*, 2009 ▸). NMR studies also found that additives such as citric acid and gypsum seeds do not change the total amount of water needed to convert hemihydrate to dihydrate (Song *et al.*, 2010 ▸).

One non-invasive probe which has widely been applied to solid-state processes is synchrotron X-ray radiation, used to study processes such as the reactions of layered double hydroxides (Williams *et al.*, 2005 ▸), metal organic framework synthesis (Wu *et al.*, 2015 ▸, 2017 ▸), or phase changes in CrTe_3_ (Hansen *et al.*, 2017 ▸). The formation of crystalline solids such as sodalite (Munn *et al.*, 1992 ▸), zeolite A (Davies *et al.*, 1997 ▸; Walton, Millange *et al.*, 2001 ▸), Co^2+^/Zn^2+^-exchanged zeolite A (Colyer *et al.*, 1995 ▸) or zinc phosphates (Rey *et al.*, 1995 ▸; Muncaster *et al.*, 2000 ▸; Wienold *et al.*, 2003 ▸; Walton, Norquist *et al.*, 2001 ▸) have all been monitored by synchrotron radiation (Pienack & Bensch, 2011 ▸). Other researchers have used this powerful tool to observe the growth of materials such as Ce_*x*_Zr_1–*x*_O_2_ (Tyrsted *et al.*, 2010 ▸), photocatalytic metal oxides (*e.g.* TiO_2_, SnO_2_), battery materials (LiFePO_4_, LiCoO_2_), and thermoelectrics (Bi_2_Te_3_, ZnO), as reviewed by Jensen *et al.* (2014 ▸). The use of high-energy synchrotron X-rays allows diffraction patterns to be collected in a few seconds, and thus reaction processes can be investigated and phase fractions accurately quantified. Synchrotron X-rays also have high intensity so even with collimation can penetrate a sample environment to allow time-resolved studies of processes under realistic laboratory conditions. This method has previously been applied to the hydro­thermal conversion of gypsum to hy­droxy­apatite, but not to the conversion of CaSO_4_·0.5H_2_O to CaSO_4_·2H_2_O (Fisher & Walton, 2009 ▸). In this work, we make use of the state-of-the-art facilities at Diamond Light Source to obtain refinement-quality patterns during the hydration of CaSO_4_·0.5H_2_O. As a result, we are able to provide unprecedented insight into the gypsum setting process.

## 2. Methods   

### 2.1. Materials   

α-CaSO_4_·0.5H_2_O, β-CaSO_4_·0.5H_2_O and ball-milled accelerant (BMA; a commercially used accelerator comprising a mixture of raw gypsum seeds and starch) were obtained from the Etex Group (Avignon, France). Water was deionized before use.

### 2.2. *Ex situ* setting experiments   

Setting experiments were performed to generate CaSO_4_·2H_2_O samples for *ex situ* analyses (see Section 2.3). 45 g of α- or β-calcium sulfate hemihydrate was added to 18 g (for α-hemihydrate) or 36 g (β-hemihydrate) of water, and mixed by hand for 1 min. Different water amounts were used in the two cases owing to differences in the water demand to finish crystallization (O’Brien, 2008 ▸). The slurry was then poured into a disc mould (height 5 cm; radius 1.5 cm; volume 35.34 cm^3^) and the mould lifted. The slurry formed a disc of around 10 cm in diameter, which was left to air dry for 24 h.

### 2.3. *Ex situ* characterization   

Infrared (IR) spectroscopy was performed in attenuated total reflectance mode using a Perkin Elmer Spectrum 100 spectrometer. Spectra were collected from 4000 to 650 cm^−1^ at a resolution of 4 cm^−1^. Scanning electron microscopy (SEM) images were recorded with the aid of an FEI Quanta FEG 200 instrument. Samples were sputter coated with gold before measurement to render them electrically conductive. Thermal analysis of the samples was performed using thermogravimetric analysis (TGA; TA Instruments Discovery instrument) and differential scanning calorimetry (DSC; TA Instruments Q2000). TGA was undertaken at a heating rate of 5°C min^−1^ under an N_2_ flow of 25 ml min^−1^. DSC thermograms were collected at 5°C min^−1^ with an N_2_ flow of 50 ml min^−1^. Experiments were performed in triplicate, and representative datasets are shown.

### 2.4. *In situ* experiments   

**Figure 1:**
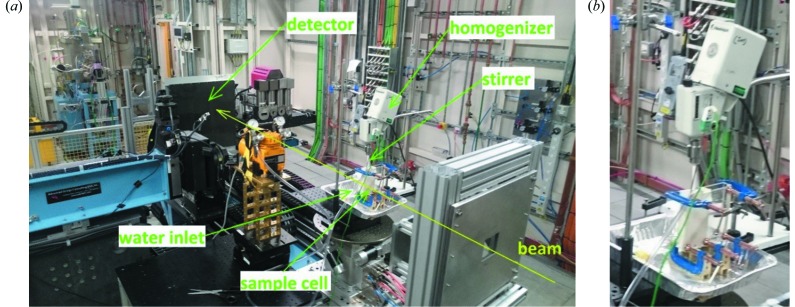
The experimental set-up on beamline I12. (*a*) The overall set up; (*b*) a close-up of the reaction vessel.

Time-resolved diffraction measurements were performed at Diamond Light Source on beamline I12 (JEEP). Monochromated X-rays were used (energy = 55.012 keV; λ = 0.2296 Å). The wavelength was calculated following a previously described protocol (Hart *et al.*, 2013 ▸). A Thales Pixium RF4343 detector was employed to collect X-ray diffraction (XRD) patterns. The distance between the detector and sample was 1635.77 mm. Bespoke apparatus was built in-house from PlexiGlas to monitor the hydration of CaSO_4_·0.5H_2_O (see Fig. S1 of the supporting information). The sample holder was assembled and loaded with CaSO_4_·0.5H_2_O (39 g for α-hemihydrate or 31.25 g for β-hemihydrate) before being placed in the experimental hutch and fitted with a Heidolph 741 overhead homogenizer (Fig. 1 ▸). Where BMA was used, it was mixed with the hemihydrate before the powder mix was placed in the sample holder. BMA acts as an accelerator to speed up the conversion time, and is widely employed to this end in industry. A pump was mounted above the sample holder, and a thermocouple inserted into the dry powder (with great care taken to avoid it interfering with stirring). A few diffraction patterns were collected of the dry powder, before the homogenizer was switched on (2000 RPM) and the pump used to dispense water (17 ml for α-hemihydrate and 25 ml for β-hemihydrate) into the sample holder to begin the hydration process. The slurry was stirred at 2000 RPM for 2 min and stirring then halted. Throughout the hydration process, XRD patterns were collected every 5 s (4.8 s collection time). Patterns were collected until they ceased to change.

Experimental data were collected as 2D Pixium images and azimuthal integration performed using *Dawn Workbench* (version 2.5.0), followed by background subtraction. The resultant patterns were then analyzed with *TOPAS Academic* (version 5), using structures reported in the Inorganic Chemistry Structural Database (ICSD). The background was fitted using Chebyshev functions, and the peak shapes with Gaussians. Rietveld refinements were undertaken using the following database entries for CaSO_4_·0.5H_2_O: 262106 (*C*2); 79529 (*I*2); 73262 (*P*3_1_21); 24474 (*P*3_2_21); and 167054 (*P*3_1_). For CaSO_4_·2H_2_O, entries 15982 (*C*2/*c*) and 36186 (*C*2/*m*) were employed. Lattice parameters were refined, and phase fractions calculated. The latter were used to determine the percentage of each phase present and the extent of the reaction.

## 3. Results   

### 3.1. Characterization of calcium sulfate phases   

#### 3.1.1. Hemihydrates   

As has widely been reported in the literature, the α- and β-CaSO_4_·0.5H_2_O materials are indistinguishable by XRD [Fig. S2(*a*)]. The hemihydrates are also similar in their IR spectra [Fig. S2(*b*)]. There are some small differences in the peak shapes in IR, but the positions are virtually identical.

The thermal data of the two materials (Fig. S3) show more distinct differences. The TGA traces clearly show that the temperature of maximum mass loss for β-CaSO_4_·0.5H_2_O is 94°C whereas for α-CaSO_4_·0.5H_2_O it is 105°C [see Fig. S3(*a*)]. Loss of 6.06% ± 0.08% of the initial mass is seen for the α-hemihydrate and 6.49% ± 0.13% for the β analogue. The difference presumably arises from surface adsorbed water, and is not thought to be significant. Both values are very close to the theoretical loss of 6.21% for the process CaSO_4_·0.5H_2_O → CaSO_4_ and the variation between them is within the error of the experiment. The later onset of water loss from α-CaSO_4_·0.5H_2_O is also observed by DSC [Fig. S3(*b*); 152.0°C for α-hemihydrate and 149.5°C for β-hemihydrate], and the dehydration endotherm for β-CaSO_4_·0.5H_2_O is broader and less intense than that for the α-form. While α-CaSO_4_·0.5H_2_O appears to show a small exotherm immediately after water loss, this is not seen for β-CaSO_4_·0.5H_2_O; the latter instead shows a small exotherm at *ca*. 365°C. The exact significance of these exothermic events is disputed in the literature (Clifton, 1972 ▸). Overall, the data are in full agreement with previous reports (Clifton, 1972 ▸; Powell & Way, 1962 ▸; Guan *et al.*, 2011 ▸; Pan *et al.*, 2013 ▸), and correspond to the water of crystallization being less firmly bound in the crystal lattice in the case of the β-hemihydrate.

There are also clear differences between the hemihydrates in terms of their crystal habit. SEM images (Fig. S4) reveal that α-CaSO_4_·0.5H_2_O has very dense-looking columnar particles of *ca.* 40 µm in length, along with some rods and more irregularly shaped particles. In contrast, the morphology of β-CaSO_4_·0.5H_2_O is much more irregular, and the secondary particle size smaller.

#### 3.1.2. Dihydrates   

The dihydrates obtained from both α- and β-CaSO_4_·0.5H_2_O (denoted α- and β-CaSO_4_·2H_2_O for clarity) appear to be identical by XRD, IR, TGA and DSC (Fig. S5). In both cases, the mass loss of approximately 21% observed in the TGA traces at *ca.* 125°C is consistent with the loss of two equivalents of water. There are, however, some differences in the crystal habits of the gypsum produced, as can be seen by SEM (Fig. S6). The α-CaSO_4_·2H_2_O material has a more densely packed network of crystals, and the primary particles are larger and more regular in shape than those in β-CaSO_4_·2H_2_O. The SEM images closely resemble those reported in other work (Carvalho *et al.*, 2008 ▸; Feng *et al.*, 2007 ▸; Wang & Meldrum, 2012 ▸), and the difference in the properties of plasters produced from the two hemihydrates is well documented. The plaster produced from α-hemihydrate has much higher mechanical strength due to different patterns of interlocking of the crystals, and is also used when fine detail in a cast is needed. Plaster from β-hemihydrate is used when lightweight material is required (Bruce *et al.*, 2012 ▸).

#### 3.1.3. BMA   

BMA is prepared from ball-milling gypsum with starch. The BMA XRD pattern [Fig. S7(*a*)] shows all the reflections for gypsum, with no clear evidence for the presence of starch. The IR spectrum shows absorption bands characteristic for gypsum [Fig. S7(*b*)], with further peaks which can be attributed to the presence of starch. In the TGA trace [Fig. S7(*c*)] there are two clear stages of mass loss. The first occurs at around 90–120°C and corresponds to water loss, while the second at 280–350°C is attributable to the decomposition of starch (Liu *et al.*, 2013 ▸). The water mass loss is around 15%, reflecting the presence of starch as well as gypsum in the BMA. SEM images (Fig. S8) demonstrate that BMA comprises spherical particles of around 2–10 µm in size.

### 3.2. Structure selection   

**Figure 2:**
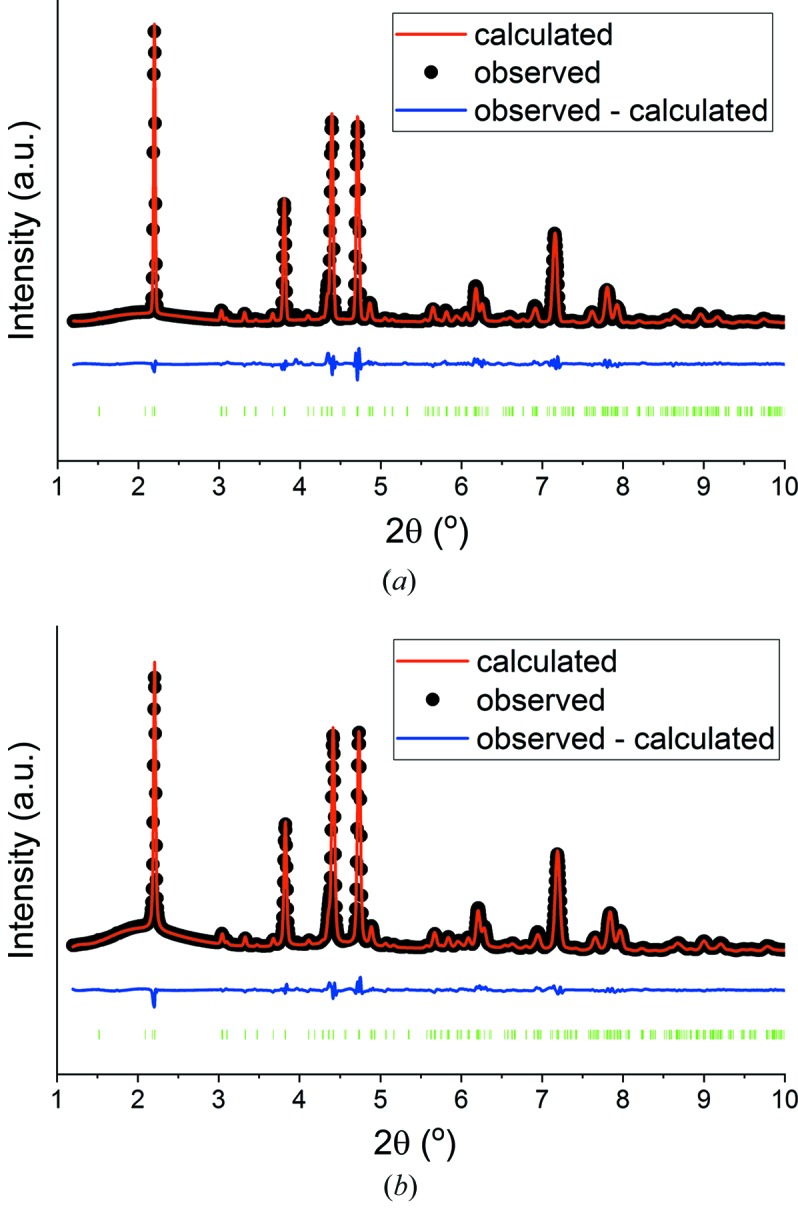
Rietveld plots for (*a*) α-CaSO_4_·0.5H_2_O and (*b*) β-CaSO_4_·0.5H_2_O in the *C*2 space group.

A number of structures have been reported in the literature for CaSO_4_·0.5H_2_O (Christensen *et al.*, 2010 ▸; Schmidt *et al.*, 2011 ▸; Bezou *et al.*, 1995 ▸; Abriel & Nesper, 1993 ▸; Gallitelli, 1933 ▸). These are all essentially the same, but propose different space groups for the structure (*C*2, *I*2, *P*3_1_, *P*3_1_21 and *P*3_2_21). To determine the best structure to use for onward analysis, *TOPAS* was employed to refine these different structures against the data obtained on I12 for the two hemihydrates in powder form. A summary of the *R*_wp_ factors obtained is given in Table 1 ▸, and full details of the refinements are presented in Table S1. The best fit is obtained in the *C*2 space group; the refinements obtained in *C*2 are presented in Fig. 2 ▸ with the remainder shown in Figs. S9 and S10.

The same fits were performed for α- and β-hemihydrates in the presence of 0.2%, 0.5% and 1% BMA (Figs. S11–S16). As would be expected, the addition of small amounts of accelerant does not make any significant difference to the refinements, and the *C*2 structure (ICSD 262106) remains appropriate for refinement (see Table S2).

**Figure 3:**
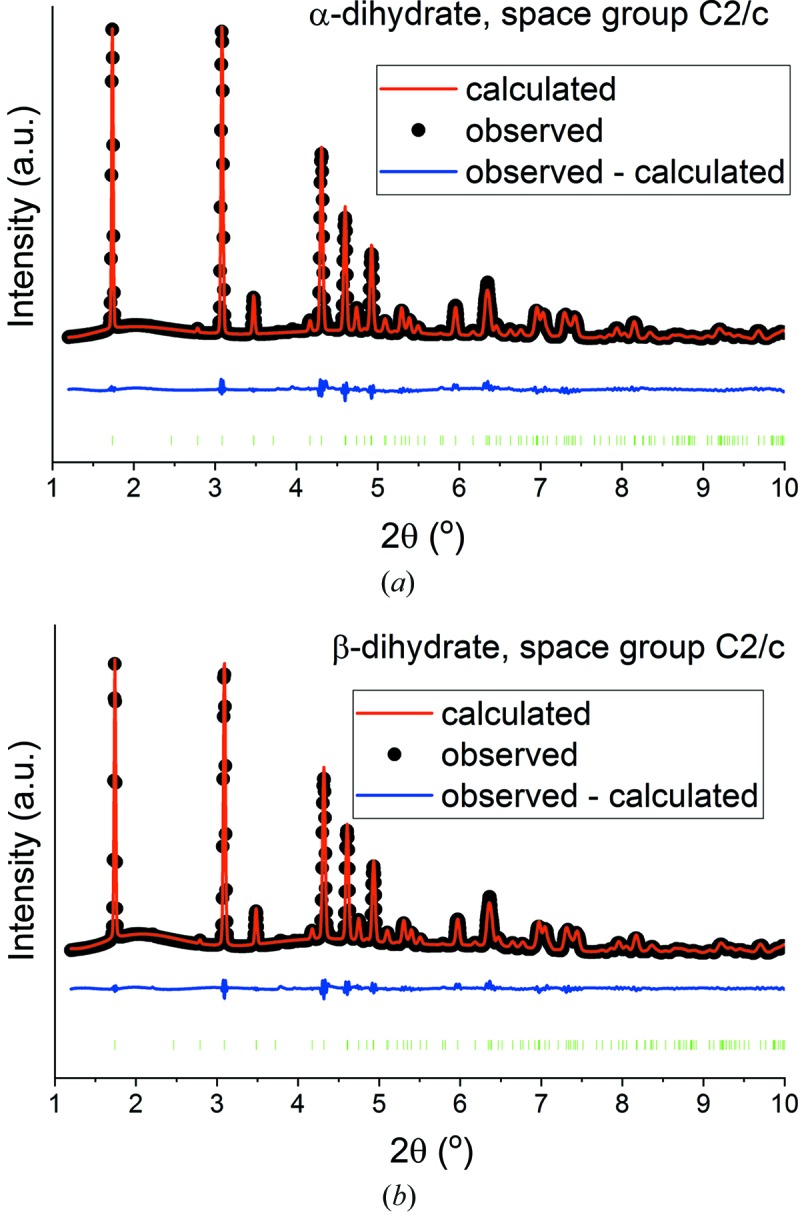
Rietveld plots for (*a*) α-CaSO_4_·2H_2_O and (*b*) β-CaSO_4_·2H_2_O using the *C*2/*c* model.

The ICSD also reports two space groups for CaSO_4_·2H_2_O [*C*2/*c* (ICSD 15982) and *C*2/*m* (ICSD 36186)]. The latter is generally agreed to be inappropriate however, and thus Rietveld refinement was performed on the products of the hydration process using the *C*2/*c* space group. The results of these fits are presented in Fig. 3 ▸.

It is very clear from Fig. 3 ▸ that very good fits are obtained with the *C*2/*c* structure, as can also be seen in the *R*_wp_ values (Table 2 ▸). Additional refinement details are listed in Table S3. No differences are observed in the products of hydration, confirming the dihydrates from both α- and β-hemihydrate to be structurally identical.

### 3.3. *In situ* diffraction studies   

**Figure 4:**
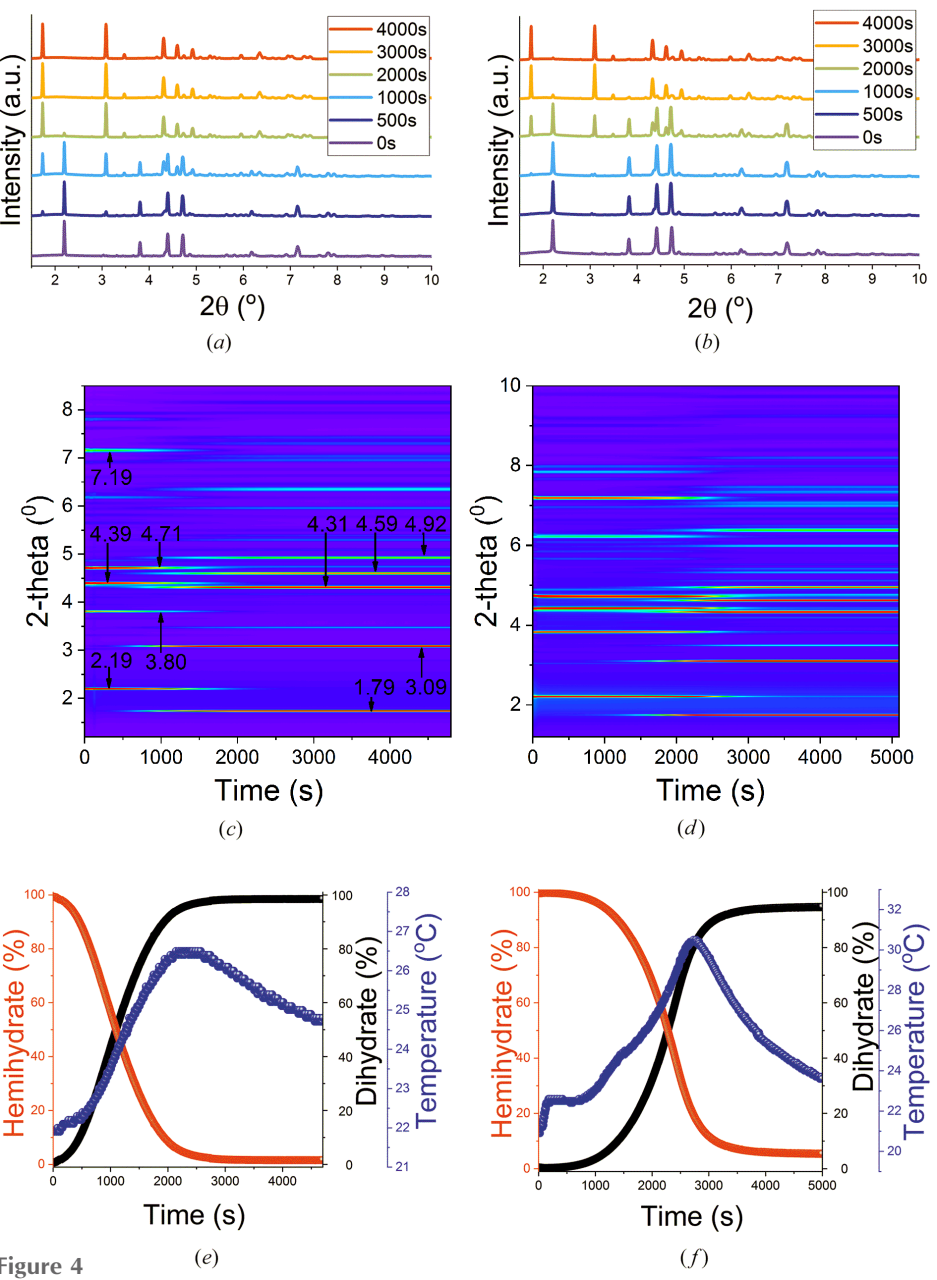
Time-resolved data showing the hydration of (*a*, *c*, *e*) α-CaSO_4_·0.5H_2_O and (*b*, *d*, *f*) β-CaSO_4_·0.5H_2_O. (*a*, *b*) Diffraction patterns obtained at selected times after the start of reaction. (*c*, *d*) Contour plots of the XRD data as a function of time. (*e*, *f*) Phase fractions of the hemi- and dihydrate determined by batch Rietveld refinements and plotted in percentage terms. The numbers in panels (*c*) and (*d*) denote the 2θ positions of the major reflections.

Kinetic data for the hydration of the hemihydrates in the absence of accelerant are given in Fig. 4 ▸. Immediately after the addition of water, no changes to the hemihydrate patterns are seen (Fig. S17). However, over time, distinct evolution in the patterns is noted (Fig. 4 ▸).

As time progresses the hemihydrate reflections at 2.19, 3.80, 4.39, 4.71 and 7.19° decline in intensity, and distinctive gypsum reflections grow in at 1.79, 3.09, 4.31, 4.59 and 4.92° [Figs. 4(*a*), 4(*b*), 4(*c*), 4(*d*) ▸]. The contour plots [Figs. 4(*c*), 4(*d*) ▸] also indicate that the reflections for gypsum appear later in time for β-hemihydrate than for α-hemihydrate, suggesting a longer induction time for the former. This has previously been reported in the literature (Song *et al.*, 2010 ▸). There are stark differences between the plots of phase fraction versus time [Figs. 4(*e*), 4(*f*) ▸]: while hydration of the α-hemihydrate begins almost immediately, there is an induction time of around 9 min before the β-form begins to react (Table 3 ▸).

For α-CaSO_4_·0.5H_2_O, 99.8% of the hemihydrate is converted to the dihydrate, while for β-hemihydrate the conversion is lower at 94.7%. It is not completely obvious why this should occur, since in both experiments sufficient water was provided to allow the hydration process to reach completion. For both α-CaSO_4_·0.5H_2_O and β-CaSO_4_·0.5H_2_O, the percentage versus time curves for the hemihydrate and dihydrate cross at the 50% point, indicating that hydration is a single-step process proceeding directly from the starting material to the product (if intermediates were present, the curves would cross at around 0%). The temperature of the system increases from around 20°C at the start of the experiment to 26.5°C or 30.5°C for the α- and β-systems, respectively. A noticeably higher temperature is thus observed in the conversion of the β-hemihydrate. The maximum temperature is reached well before the hydration process is complete (at 93.9 and 80.4% for the α- and β-systems, respectively). This is important because in industrial research and development temperature is often used as a pr­oxy for the extent of hemihydrate hydration: these data show that it is a poor surrogate measure, and alternatives should be explored.

**Figure 5:**
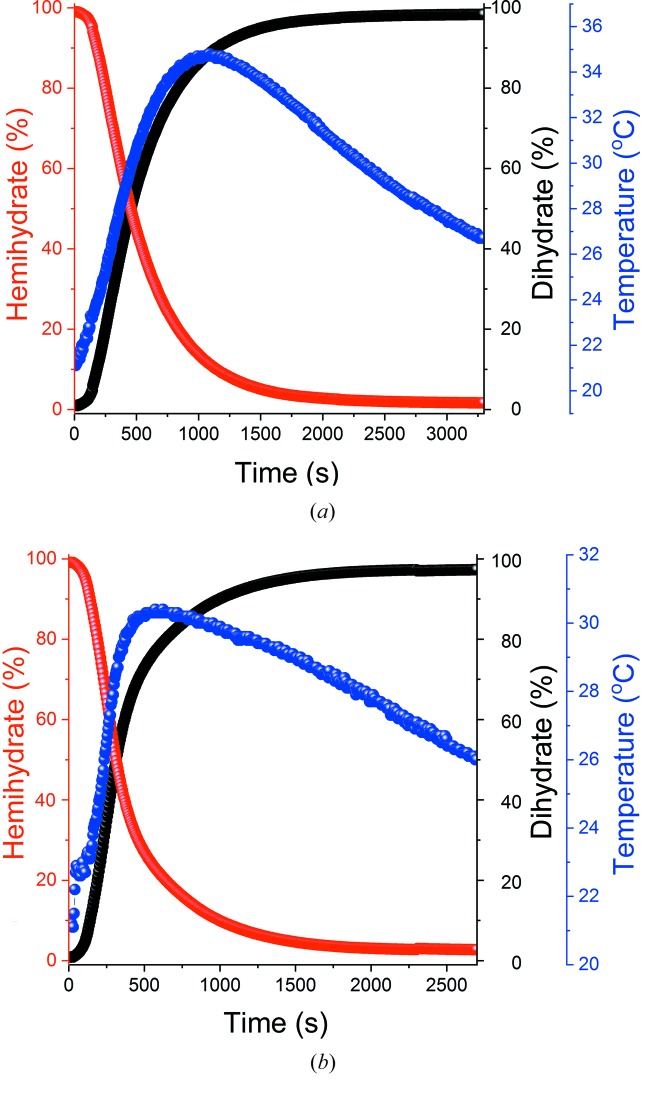
*In situ* time-resolved data for the hydration of (*a*) α- and (*b*) β-CaSO_4_·0.5H_2_O in the presence of 1% *w*/*w* BMA. Phase fractions of the hemi- and dihydrate were determined by batch Rietveld refinements, and are plotted in percentage terms.

**Figure 6:**
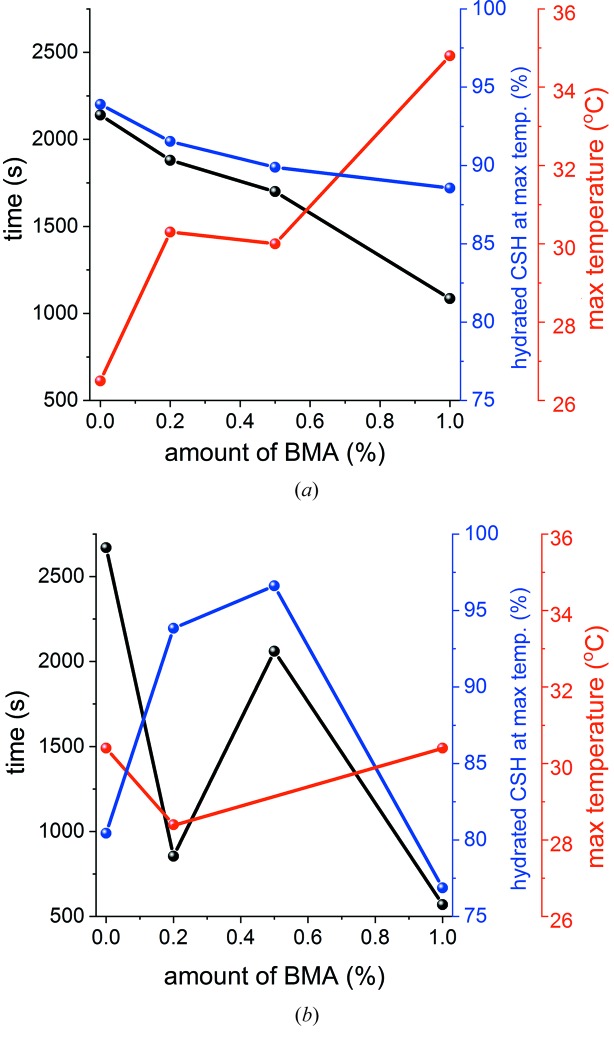
Temperature data for (*a*) α-hemihydrate and (*b*) β-hemihydrate hydration, showing the time taken to reach the maximum temperature and the percentage conversion at which the temperature peaks. CSH denotes CaSO_4_·0.5H_2_O.

The conversion process from hemi- to dihydrate was also observed *in situ* for both α-CaSO_4_·0.5H_2_O and β-CaSO_4_·0.5H_2_O in the presence of BMA as an accelerator. Different concentrations of 0.2, 0.5 and 1% BMA (*w*/*w*, with respect to the mass of hemihydrate) were explored. Data for 1% *w*/*w* BMA are presented in Fig. 5 ▸, with the remaining data in Fig. S18. The addition of BMA causes the reaction to proceed more quickly, and the maximum temperature to increase in the case of α-CaSO_4_·0.5H_2_O, with top temperatures of 35°C reached with 1% BMA (*cf.* 26.5°C without BMA). The maximum temperature is also reached at lower conversion percentages as the amount of BMA increases [see Fig. 6(*a*) ▸]. The temperature change is more complex in the case of β-CaSO_4_·0.5H_2_O, with the highest temperatures attained lying in the region of 28.5–30°C. It appears that intermediate amounts of BMA result in the smallest temperature increase, and this being reached at the greatest conversion percentage [Fig. 6(*b*) ▸]. The maximum temperature generally appears to be reached at higher conversion percentages for α-CaSO_4_·0.5H_2_O than β-CaSO_4_·0.5H_2_O.

A series of plots were constructed to evaluate the variation in unit-cell parameters and cell volume of the hemi- and dihydrate phases during the hydration process (data not shown). No significant changes were observed.

### 3.4. Kinetic modelling   

**Figure 7:**
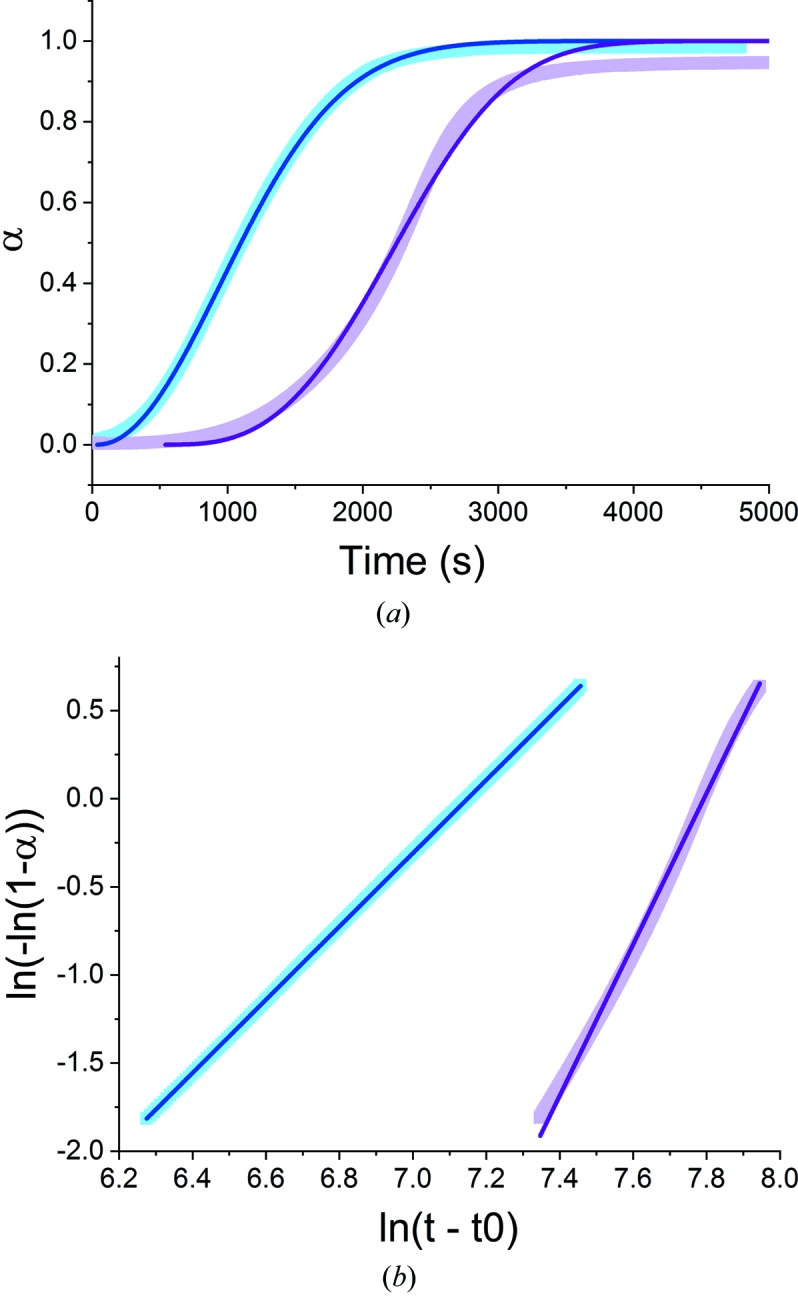
The results of fitting the Avrami–Erofe’ev model to the hydration of CaSO_4_·0.5H_2_O, showing (*a*) the extent of the reaction versus time and (*b*) Sharp–Hancock plots for (cyan) α- and (magenta) β-CaSO_4_·0.5H_2_O hydration.

For the application of kinetic models, the conversion percentage was first normalized to give the extent of the reaction, α (Kennedy & Clark, 1997 ▸). Attempts were then made to fit a series of well known kinetic models (Table S4) to the data. In general, the fits were poor (see Table S5 and Figs. S19 and S20). The best fits were observed with the Avrami–Erofe’ev and Gualtieri models, which result in the closest visual fits and highest *R* ^2^ values. The Avrami–Erofe’ev model [equation (1)] is widely used to describe solid-state transformations (Avrami, 1940 ▸, 1941 ▸),
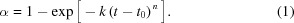
α is the extent of the reaction, *n* a reaction exponent which gives information on the mechanism of the reaction, *k* the rate constant, *t* the elapsed time and *t*_0_ the induction time. This equation is valid over the range 0.15 < α < 0.85 (Du *et al.*, 2008 ▸), and can conveniently be rearranged to give
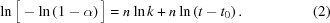
If ln[−ln(1 − α)] is plotted against ln *t* (a Sharp–Hancock plot), a linear graph will result if the model is valid for the system being studied. Fits of the Avrami–Erofe’ev equation to the hydration of CaSO_4_·0.5H_2_O in the absence of accelerant are given in Fig. 7(*a*) ▸ and Sharp–Hancock plots in Fig. 7(*b*) ▸. For α-CaSO_4_·0.5H_2_O the fits are good, but the Sharp–Hancock plots in particular reveal distinct non-linearity for β-CaSO_4_·0.5H_2_O, indicating that the Avrami–Erofe’ev model is not suitably complex to model this process.

Similar results are obtained when BMA was used to speed up the reaction (Fig. S21). The Avrami fits are noticeably better for α-CaSO_4_·0.5H_2_O, and the Sharp–Hancock plots are highly linear. However, in the β-CaSO_4_·0.5H_2_O case the Avrami fits are less close, and distinctly non-linear Sharp–Hancock plots are seen. This deviation from linearity increases with the amount of BMA used. The values of *n* and *k* extracted from the Sharp–Hancock plots are given in Table 4 ▸. The rate of reaction, *k*, tends to increase with the BMA concentration; *n* is also generally seen to decrease with the amount of BMA added. Hulbert has analysed in detail the possible *n* values which may be derived from the Avrami–Erofe’ev equation (Hulbert, 1969 ▸), but in this case it is not possible to unambiguously determine the mechanism of reaction since the values of *n* seen could indicate multiple possibilities. The decline in the value of *n* with increasing BMA is, however, consistent with a reduced importance of nucleation in determining the rate of reaction, which is sensible given the increased number of nucleation sites present with more BMA.

**Figure 8:**
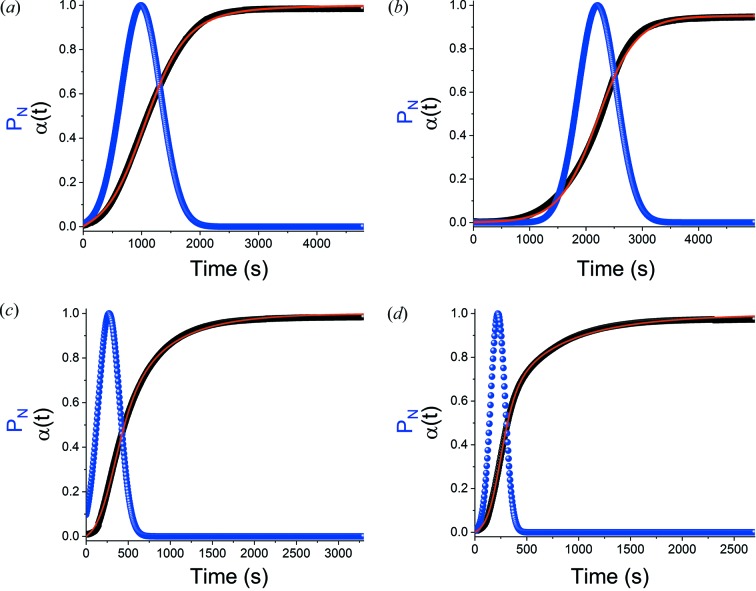
Gualtieri fits for the hydration of (*a*) α-CaSO_4_·0.5H_2_O; (*b*) β-CaSO_4_·0.5H_2_O; (*c*) α-CaSO_4_·0.5H_2_O with 1% *w*/*w* BMA; and (*d*) β-CaSO_4_·0.5H_2_O with 1% *w*/*w* BMA. Experimental data (filled black squares), the corresponding Gualtieri fits (red line) and the calculated rate of nucleation (*P*_N_; blue circles) are depicted.

The best fit to the experimental data was obtained with the Gualtieri model (Gualtieri, 2001 ▸). This expresses the crystal growth process as detailed in equation (3),
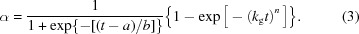

*t* is the reaction time, *a* and *b* are parameters related to the nucleation process, *k*_g_ is the rate of crystal growth, and *n* is the dimensionality of growth. SEM images (Fig. S6) indicate that the crystal habit is needle-like, and hence *n* was set to 1 for this analysis. The *b* parameter contains information about the crystal growth mechanism, while *a* is closely related to the rate of nucleation, *k*_*n*_ [equation (4)] (Etampawala *et al.*, 2016 ▸),
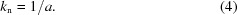

*a* and *b* can also be used to determine the probability of nucleation, *P*_N_ [equation (5)] (El Osta *et al.*, 2013 ▸),
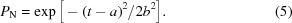
Gualtieri fits for the reactions undertaken without BMA and with 1% *w*/*w* BMA are depicted in Fig. 8 ▸ (the data for 0.2 and 0.5% *w*/*w* BMA are given in Fig. S22).

As was observed with the Avrami–Erofe’ev model, the fits are closer for α-CaSO_4_·0.5H_2_O than for β-CaSO_4_·0.5H_2_O, but in general the *R* ^2^ values are high and visibly it can be seen that the fits are all good. A summary of the kinetic parameters extracted is shown in Table 5 ▸.

**Figure 9:**
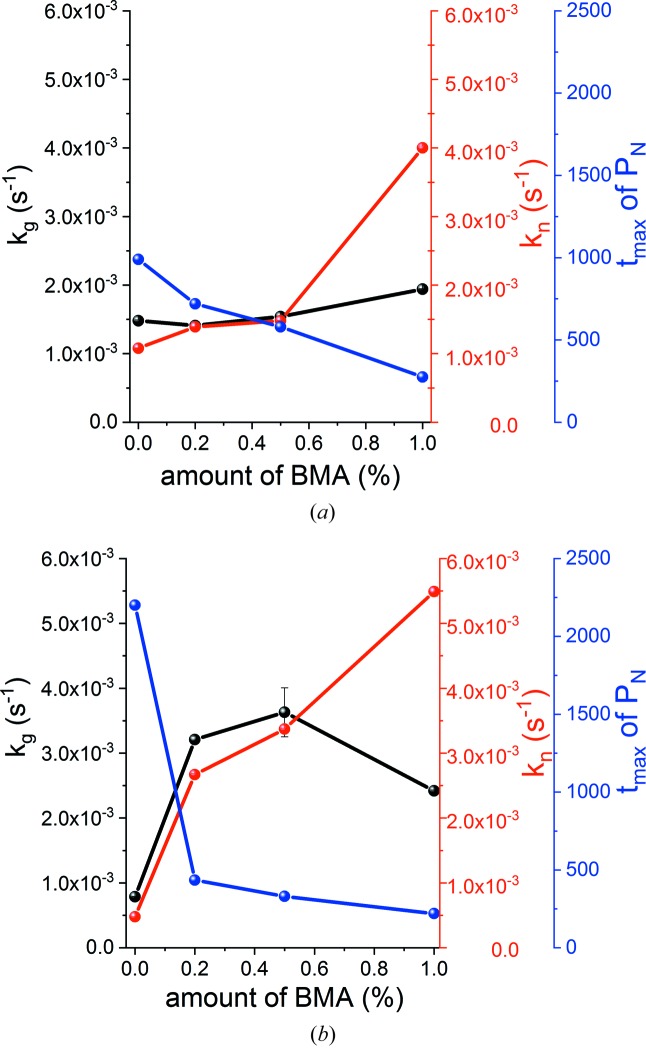
Crystal growth rate, rate of nucleation and the time at which the probability of nucleation is greatest as a function of BMA concentration: (*a*) α-hemihydrate; (*b*) β-hemihydrate.

The first key finding (Table 5 ▸) is that the addition of BMA generally causes both the rate of nucleation (*k*_n_) and rate of crystal growth (*k*_g_) to increase. The trends are not completely continuous with *k*_g_, and the increases in *k*_n_ are much more consistent and marked (Fig. 9 ▸). *k*_g_ is greater than *k*_n_ for systems where the BMA concentration is low (up to 0.5% for both α- and β-CaSO_4_·0.5H_2_O). This indicates that nucleation is the rate-limiting process where small amounts of BMA are present (Etampawala *et al.*, 2016 ▸). The difference between *k*_g_ and *k*_n_ narrows as the amount of BMA present increases, indicating a decrease in the importance of nucleation in controlling the reaction rate. In systems where the BMA concentration is increased to 1%, *k*_n_ becomes more than twofold greater than *k*_g_, and the rate-limiting process is crystal growth. This agrees with the findings from Avrami–Erofe’ev analysis.

Second, it is clear from Fig. 8 ▸ that the probability of nucleation (*P*_N_) increases rapidly for the first 990 s (16.5 min) for pure α-hemihydrate, while it reaches a maximum after 2200 s (37 min) for β-hemihydrate. Subsequently, a sharp decrease in *P*_N_ is observed in both cases. The same general trend is observed regardless of the amount of BMA present, but an increase in BMA concentration results in *P*_N_ increasing more rapidly and reaching a maximum at an earlier time point (Fig. 9 ▸).

The *b* parameter provides additional information on the nucleation process. If *b* ≤ 900 s (15 min) then the nucleation process is heterogeneous, if *b* > 1200 s (20 min) the nucleation is autocatalytic and if *b* ≃ 900 the nucleation is homogeneous (Gualtieri, 2001 ▸; Etampawala *et al.*, 2016 ▸; El Osta *et al.*, 2013 ▸; Bueken *et al.*, 2017 ▸). In all cases (Table 5 ▸), *b* is much smaller than 900 s (15 min), indicating that nucleation is heterogeneous. This is suggestive of a situation in which crystallization takes place at preformed aggregates in the reaction matrix, which is sensible for the hydration of CaSO_4_·0.5H_2_O given that the hydration process will occur at the surfaces of existing particles. The value of *b* declines notably with an increasing amount of BMA added, which is consistent with this hypothesis.

## 4. Discussion   

Using beamline I12 (JEEP) at Diamond Light Source we have been able to provide unprecedented insight into the hydration of α- and β-CaSO_4_·0.5H_2_O to CaSO_4_·2H_2_O. We show this to be a one-step process, with the phase percentage versus time curves for the starting material and product crossing at *ca.* 50%. Our data are fully consistent with the conversion from CaSO_4_·0.5H_2_O to CaSO_4_·2H_2_O following a single-step dissolution–precipitation mechanism as reported in the literature (Amathieu & Boistelle, 1986 ▸, 1988 ▸; Wang *et al.*, 2012 ▸; Singh & Middendorf, 2007 ▸). The addition of CaSO_4_·2H_2_O seeds to accelerate the process does not change the mechanism. We find that hydration is never fully completed, even if enough water is provided to drive the reaction to 100% conversion. The maximum temperature of the reaction system is reached before the end of hydration, and can occur as early as 80% conversion from hemi- to dihydrate. This is crucial information for plasterboard production, where temperature is often used as a pr­oxy measurement for hydration.

The Gualtieri kinetic model is found to provide the best fit to the experimental data for CaSO_4_·0.5H_2_O hydration in all cases. This model has been used to describe other crystallization reactions in aqueous media such as the synthesis of zeolitic imidazolate frameworks (Cravillon *et al.*, 2012 ▸), metal organic frameworks (El Osta *et al.*, 2013 ▸), ytterbium di­carboxyl­ate frameworks (Breeze *et al.*, 2017 ▸) or zeolites (Gualtieri, 2001 ▸), and the growth of silver metal-organic nanotubes (Etampawala *et al.*, 2016 ▸). All these reports find the value of the *b* factor to be lower than 15 min, consistent with heterogeneous nucleation, as noted for the hydration reaction explored in this work. The *k*_n_ and *k*_g_ values we find here lie within the range reported by previous studies (0.317 × 10^−3^ < *k*_n_ < 12 × 10^−3^ s^−1^ and 0.00567 × 10^−3^ < *k*_g_ < 16500 × 10^−3^ s^−1^). The literature also generally shows an increase in both *k*_n_ and *k*_g_ with the temperature of the reaction (Gualtieri, 2001 ▸; El Osta *et al.*, 2013 ▸; Cravillon *et al.*, 2012 ▸). Here, we see a continuous increase in the nucleation rate, *k*_n_, with the amount of BMA present. This is consistent with the reduced importance of nucleation in controlling reaction rate implied from fits of the Avrami–Erofe’ev model to the data. There is also a general tendency for *k*_g_, the rate of crystal growth, to rise with increasing BMA concentrations, but this is not universal. We should note that, while these models provide useful insight into the reaction process, both have some limitations, since they are designed for systems where crystallization takes place in a homogeneous medium that contains the species required for nucleation. There will inevitably be some degree of heterogeneity in the water/CaSO_4_·0.5H_2_O system, and thus the models could miss some additional complexity.

There are some kinetic data in the literature on the hydration of inorganics. For example, the hydration of calcium phosphate cements (used for filling non-load-bearing bone defects) was studied by Gao *et al.* (2006 ▸). The kinetics of this process were found to follow 3D diffusion (Jander) kinetics, with hydration mostly controlled by surface dissolution, three-dimensional diffusion and calcium phosphate cement nucleation. This model was not found to provide a good fit for the Ca sulfate data in this work, showing that the change in anion causes a very significant change in the reaction mechanism.

Both the crystal growth and nucleation rate are of great importance in the conversion from calcium sulfate hemihydrate to dihydrate, which forms the crux of a multi-billion pound industry. Through the unprecedented new understanding of this process delivered in our work, manufacturers have the potential to lower significantly the cost of plasterboard production.

## 5. Conclusions   

A detailed study of the hydration of CaSO_4_·0.5H_2_O using time-resolved synchrotron X-ray diffraction is reported in this work. We see very distinct differences between the α- and β-forms of CaSO_4_·0.5H_2_O. While the CaSO_4_·2H_2_O product is very similar regardless of which form of the hemihydrate is used as the starting material, differing only in its crystal habit, the kinetics and mechanism of the process are very different. In general, the hydration of α-CaSO_4_·0.5H_2_O has a shorter induction time than β-CaSO_4_·0.5H_2_O, reaches a greater conversion percentage, and leads to a wider fluctuation in temperature. The latter is found to be a poor pr­oxy for the extent of reaction, with the maximum temperature reached well before the reaction is complete. Fitting kinetic models to the time-resolved data revealed that the Avrami–Erofe’ev and Gualtieri models provide the best description of the experimental findings. The fits were substantially closer for the hydration of α-CaSO_4_·0.5H_2_O. In the Avrami analysis the rate of reaction tends to increase with the amount of gypsum seeds added to accelerate the process, and the importance of nucleation in determining the rate declines. The Gualtieri modelling revealed that the rate of nucleation increases substantially with the amount of seeds added, while there are less distinct changes in the rate of crystal growth. At low seed concentrations (<0.5% *w*/*w*) the rate of crystal growth is greater than the rate of nucleation, but this situation reverses at concentrations above 0.5% *w*/*w*.

## Supplementary Material

Supporting information containing all supporting figures: Figure S1 – Figure S22 and supporting tables: Table S1 - Table S5. DOI: 10.1107/S1600577519001929/co5118sup1.pdf

## Figures and Tables

**Table 1:** *R*_wp_ factors obtained when refining the various reported structures for CaSO_4_·0.5H_2_O against the experimental data obtained on I12 with α- and β-hemihydrate

	Space group				
	*C*2	*I*2	*P*3_1_	*P*3_2_21	*P*3_1_21
Reference	Schmidt *et al.* (2011 ▸)	Bezou *et al.* (1995 ▸)	Christensen *et al.* (2010 ▸)	Gallitelli (1933 ▸)	Abriel & Nesper (1993 ▸)
ICSD number	262106	79529	167054	24474	73262
α-CaSO_4_·0.5H_2_O	4.737	7.660	20.146	35.400	36.640
β-CaSO_4_·0.5H_2_O	3.868	4.581	25.444	39.949	40.311

**Table 2:** *R*_wp_ factors for α- and β-dihydrate fitted with structures in the *C*2/*c* model (ICSD reference: 15982; Wooster, 1936 ▸)

Sample	*R*_wp_
α-CaSO_4_·2H_2_O	5.139
β-CaSO_4_·2H_2_O	5.211

**Table 3:** Induction time (*t*_0_) values for the α- and β-hemihydrate systems, with and without BMA

Sample	*t*_0_ (s)†	Sample	*t*_0_ (s)
α-CaSO_4_·0.5H_2_O	33.6 ± 8.3	β-CaSO_4_·0.5H_2_O	539.4 ± 14.2
α-CaSO_4_·0.5H_2_O + 0.2% BMA	165.5 ± 9.4	β-CaSO_4_·0.5H_2_O + 0.2% BMA	118.6 ± 19.4
α-CaSO_4_·0.5H_2_O + 0.5% BMA	99.2 ± 7.4	β-CaSO_4_·0.5H_2_O + 0.5% BMA	176.1 ± 6.1
α-CaSO_4_·0.5H_2_O + 1% BMA	142.1 ± 2.2	β-CaSO_4_·0.5H_2_O + 1% BMA	156.1 ± 1.4

† *t*_0_ was determined by fitting the Avrami–Erofe’ev kinetic model [equation (1)] to the experimental data.

**Table 4:** Kinetic parameters calculated from Sharp–Hancock plots

Sample	*k* × 10^−5^ (s^−1^)	*n*	*R* ^2^
α-CaSO_4_·0.5H_2_O	0.0354 ± 1.72 × 10^−4^	2.08 ± 6.9 × 10^−4^	0.9999
α-CaSO_4_·0.5H_2_O + 0.2% BMA	1.25 ± 3.91 × 10^−3^	1.64 ± 4.7 × 10^−4^	0.9999
α-CaSO_4_·0.5H_2_O + 0.5% BMA	0.855 ± 3.91 × 10^−3^	1.69 ± 6.8 × 10^−4^	0.9999
α-CaSO_4_·0.5H_2_O + 1% BMA	150 ± 1.35	1.07 ± 0.001	0.9999

β-CaSO_4_·0.5H_2_O	2.27 × 10^−6^ ± 2.72 × 10^−7^	3.24 ± 0.02	0.9937
β-CaSO_4_·0.5H_2_O + 0.2% BMA	0.608 ± 0.0547	2.02 ± 0.02	0.9955
β-CaSO_4_·0.5H_2_O + 0.5% BMA	49.1 ± 4.42	1.41 ± 0.02	0.9908
β-CaSO_4_·0.5H_2_O + 1% BMA	1660 ± 66.4	0.74 ± 0.007	0.9886

**Table 5:** Gualtieri kinetic parameters calculated for the hydration of α- and β-CaSO_4_·0.5H_2_O

Sample	*a* (s)	*b* (s)	*k*_g_ × 10^−3^ (s^−1^)	*k*_n_ × 10^−3^ (s^−1^)	*R* ^2^
α-CaSO_4_·0.5H_2_O	922 ± 3.3	353 ± 1.9	1.48 ± 0.0125	1.08 ± 3.83 × 10^−3^	0.9993
α-CaSO_4_·0.5H_2_O + 0.2% BMA	718 ± 2.5	293 ± 2.1	1.41 ± 7.71 × 10^−3^	1.39 ± 4.80 × 10^−3^	0.9992
α-CaSO_4_·0.5H_2_O + 0.5% BMA	674 ± 2.4	310 ± 1.9	1.54 ± 8.77 × 10^−3^	1.48 ± 5.31 × 10^−3^	0.9994
α-CaSO_4_·0.5H_2_O + 1% BMA	250 ± 1.8	115 ± 1.7	1.94 ± 5.63 × 10^−3^	4.00 ± 0.0288	0.9988

β-CaSO_4_·0.5H_2_O	2090 ± 3.9	329 ± 3.0	0.787 ± 6.96 × 10^−3^	0.478 ± 0.0897	0.9972
β-CaSO_4_·0.5H_2_O + 0.2% BMA	375 ± 2.7	91.4 ± 2.3	3.21 ± 0.0540	2.67 ± 0.0195	0.9920
β-CaSO_4_·0.5H_2_O + 0.5% BMA	297 ± 1.6	61.7 ± 1.4	3.63 ± 0.378	3.37 ± 0.0178	0.9961
β-CaSO_4_·0.5H_2_O + 1% BMA	182 ± 2.4	50.0 ± 2.1	2.42 ± 0.0126	5.49 ± 0.0714	0.9942
