# Supplementary material for: A kinetic and mechanistic study into the transformation of calcium sulfate hemihydrate to dihydrate
Source: J Synchrotron Radiat. 2019 Apr 5;26(Pt 3):774–84. doi: 10.1107/S1600577519001929 (PMC6510200; doi:10.1107/S1600577519001929)
Supplement: Supplementary file 1 [file s-26-00774-sup1.pdf]

JOURNAL OF SYNCHROTRON RADIATION

**Volume 26 (2019)**

**Supporting information for article:**

# A kinetic and mechanistic study into the transformation of calcium sulfate hemihydrate to dihydrate

**Sebastian J. Gurgul, Gareth R. Williams and Gabriel Seng**

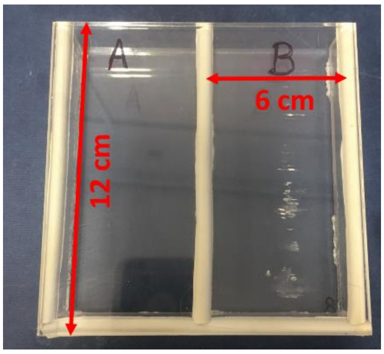


**Figure S1** The design of the in-house rig used for *in situ* diffraction studies. The depth is 1 cm.

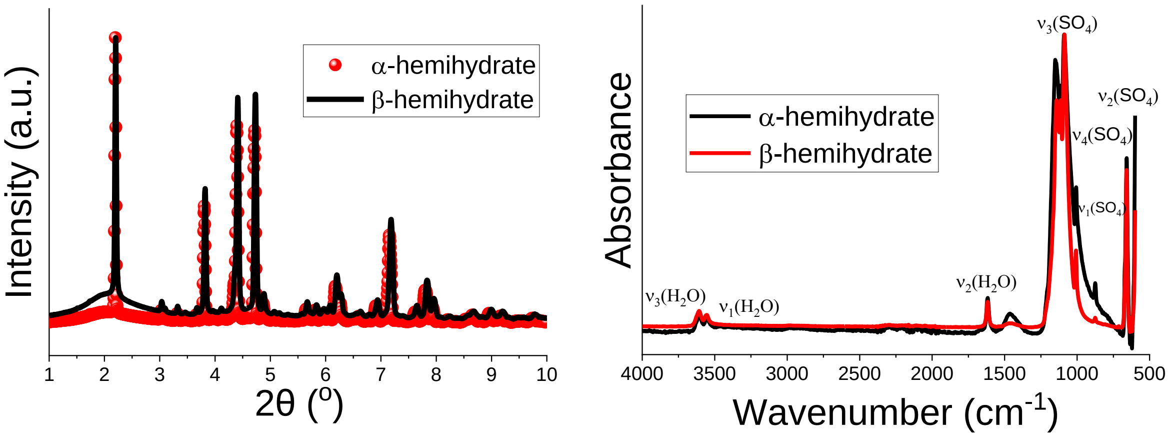


(a) (b)

**Figure S2** (a) XRD and (b) IR data for α- and β-$CaSO_4 \cdot 0.5H_2O$.

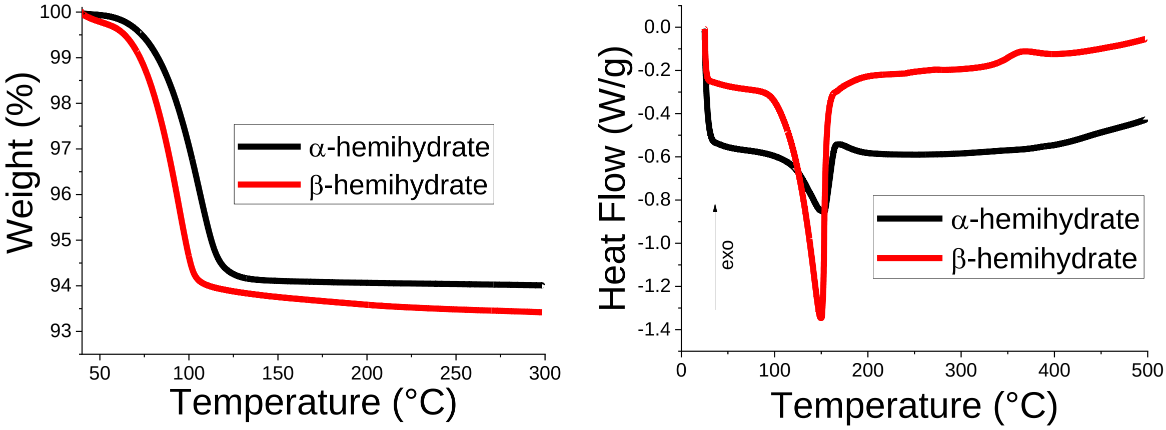


(a) (b)

**Figure S3** (a) TGA and (b) DSC data for α- and β-$CaSO_4 \cdot 0.5H_2O$.

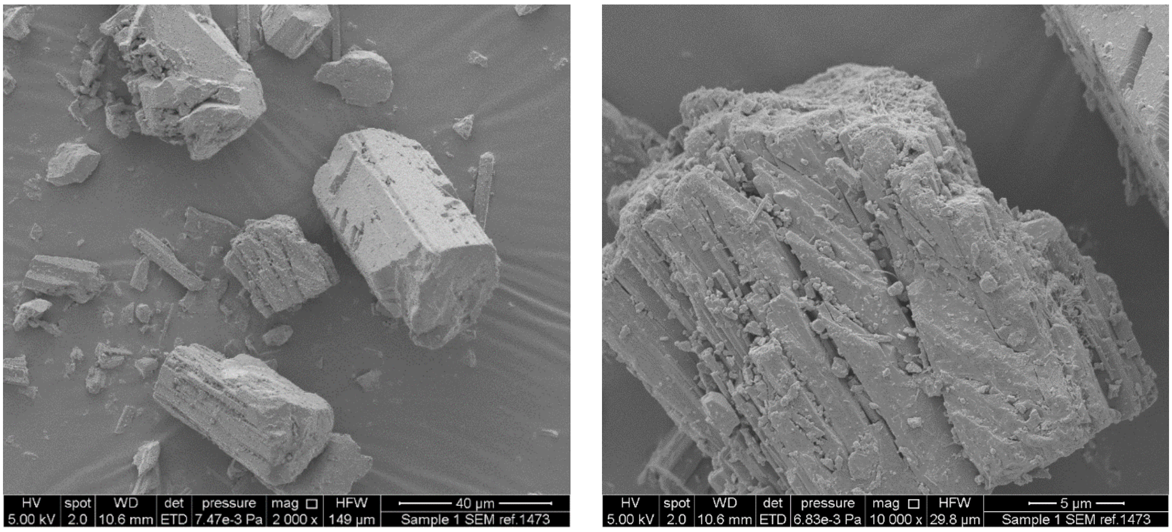


(a)

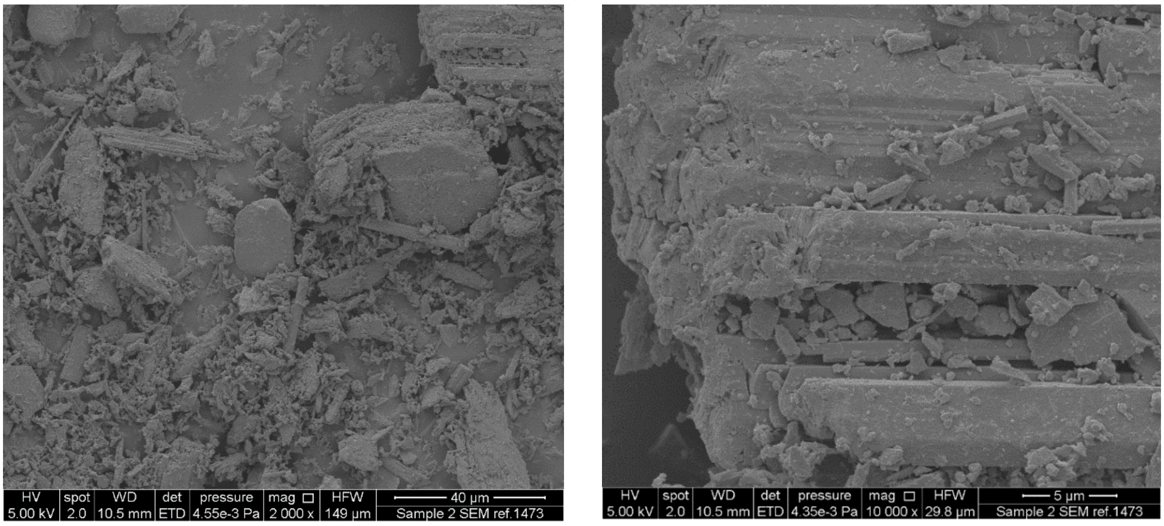


(b)

**Figure S4** SEM images of (a) α- and (b) β-$CaSO_4 \cdot 0.5H_2O$.

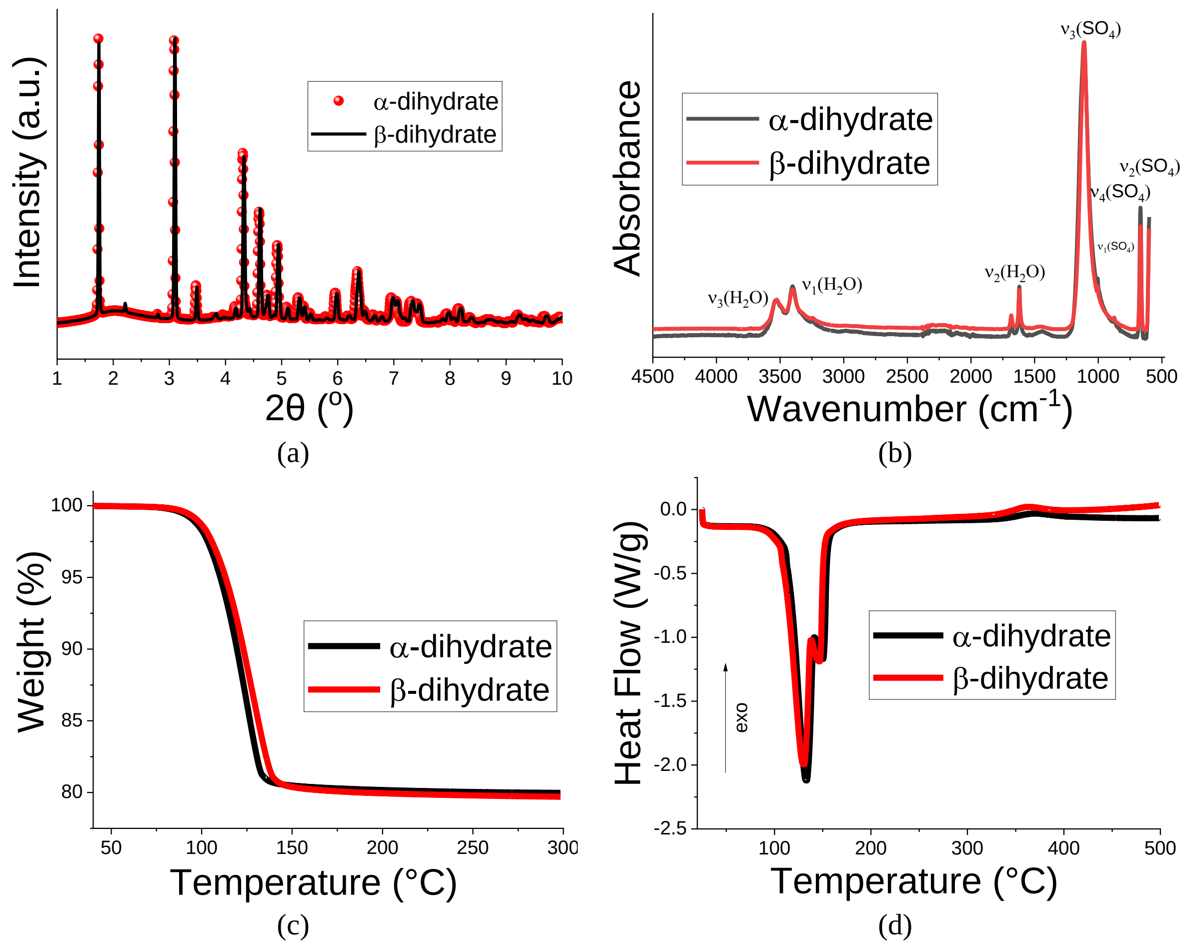


**Figure S5** Data for dihydrates obtained from α- and β-$CaSO_4 \cdot 0.5H_2O$. (a) XRD; (b) IR: (c) TGA; and, (d) DSC data are presented.

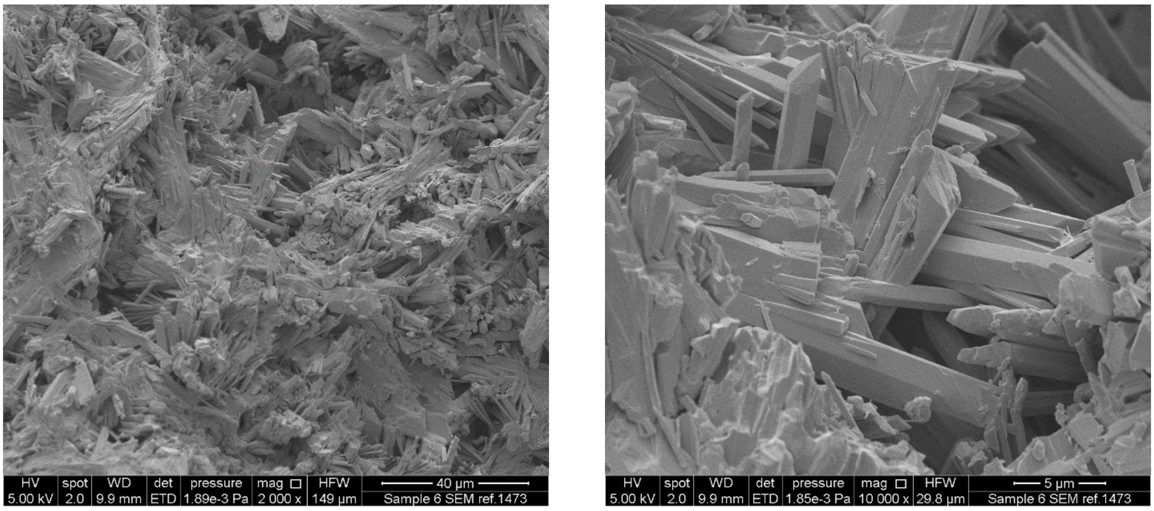


(a)

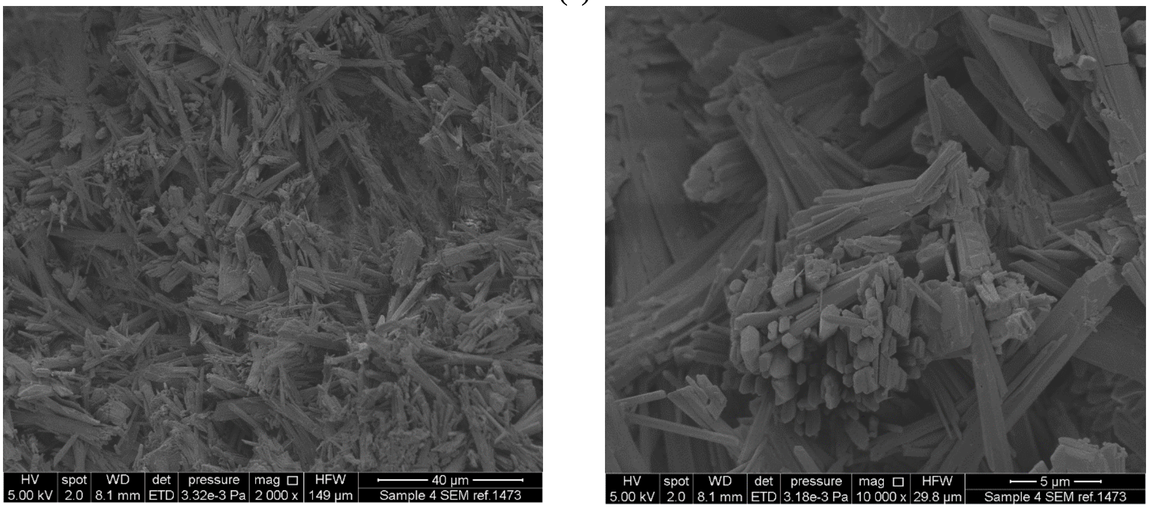


(b)

**Figure S6** SEM images of (a) α- and (b) β-$CaSO_4·2H_2O$.

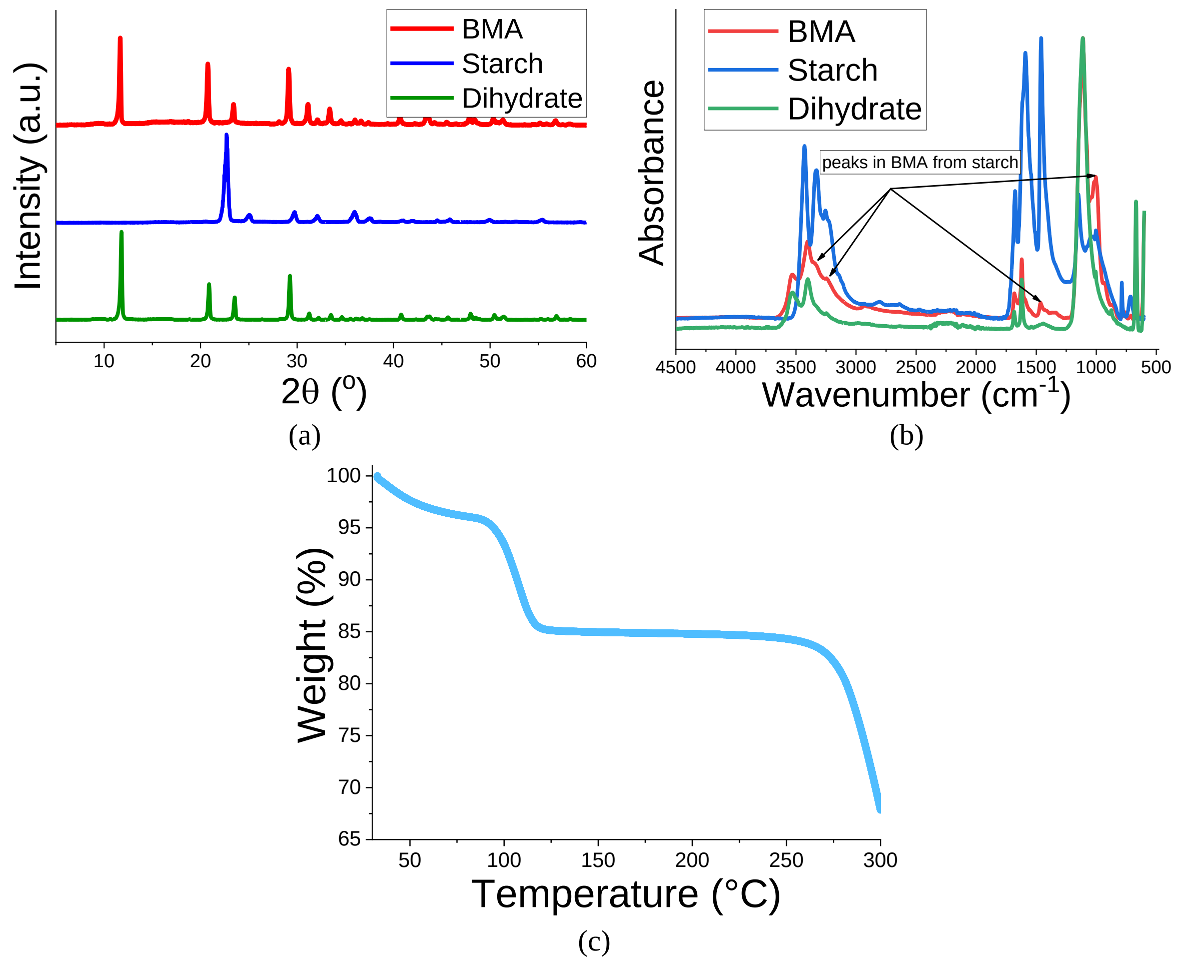


**Figure S7** (a) XRD; (b) IR; and, (c) TGA data for BMA.

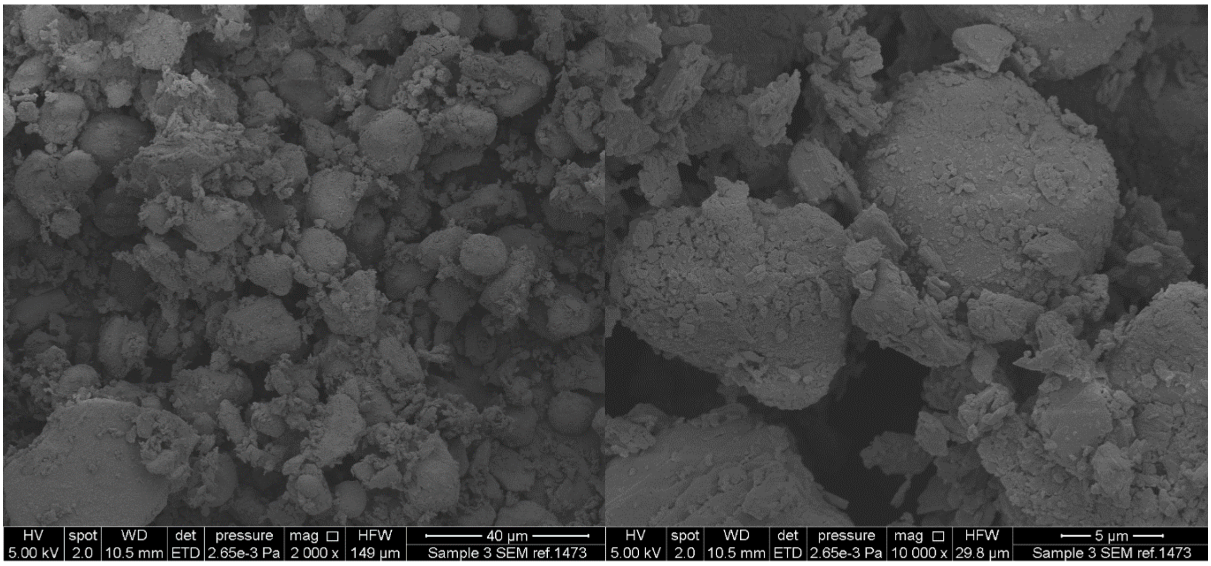


**Figure S8** SEM images of BMA.

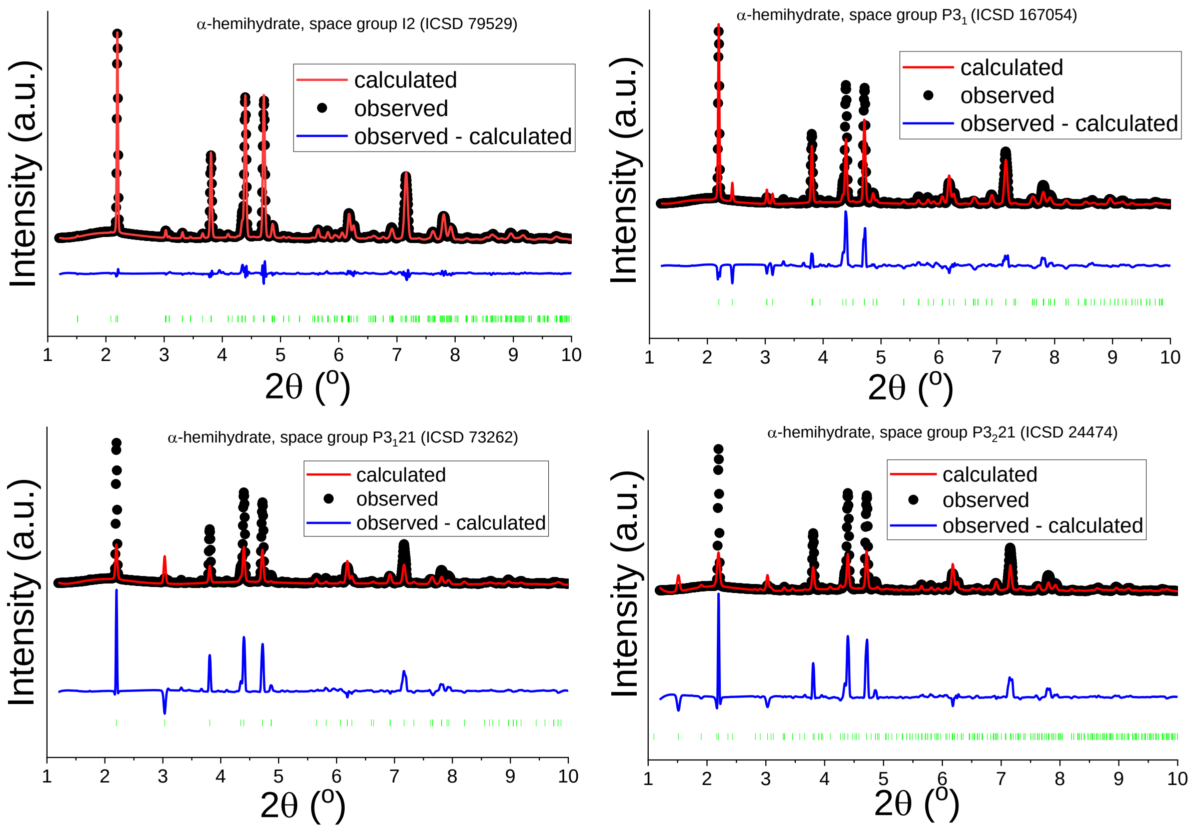


**Figure S9** The results of structural refinements with the different models reported in the ICSD for α-$CaSO_4 \cdot 0.5H_2O$.

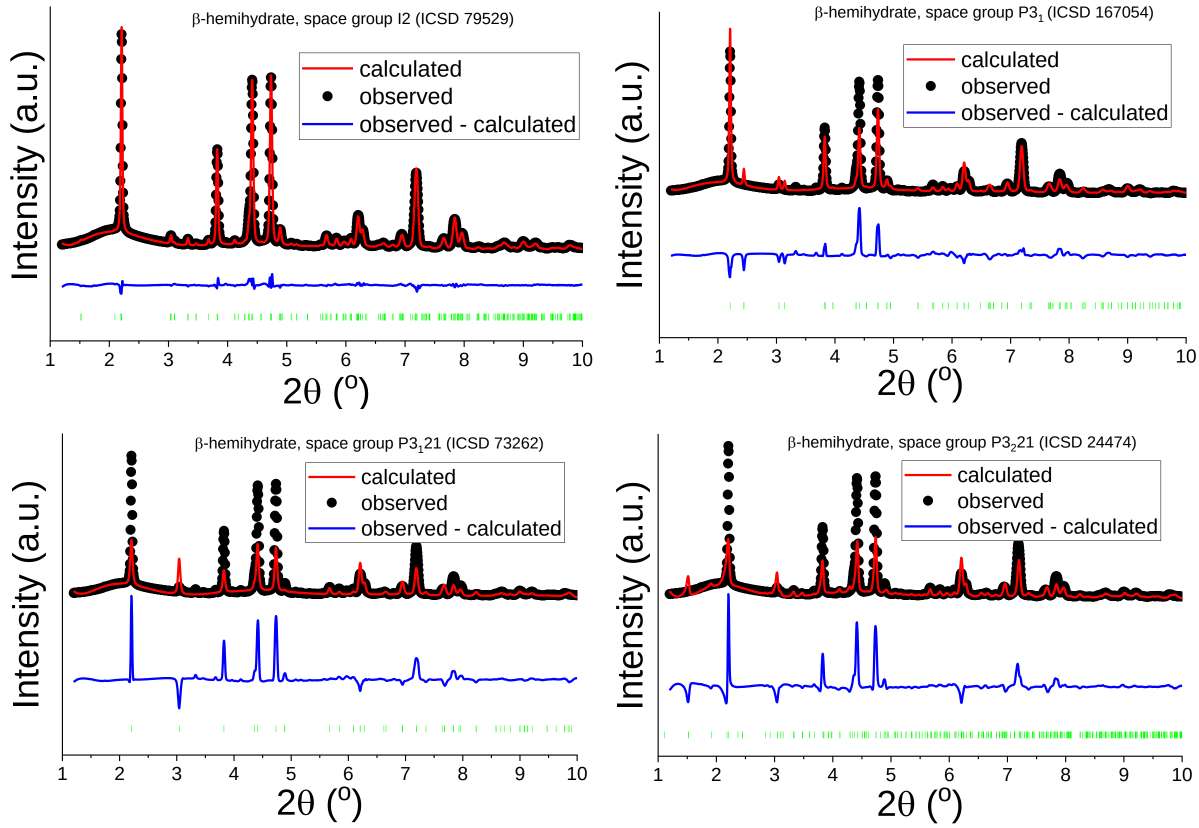


**Figure S10** The results of structural refinements with the different models reported in the ICSD for β-$CaSO_4 \cdot 0.5H_2O$.

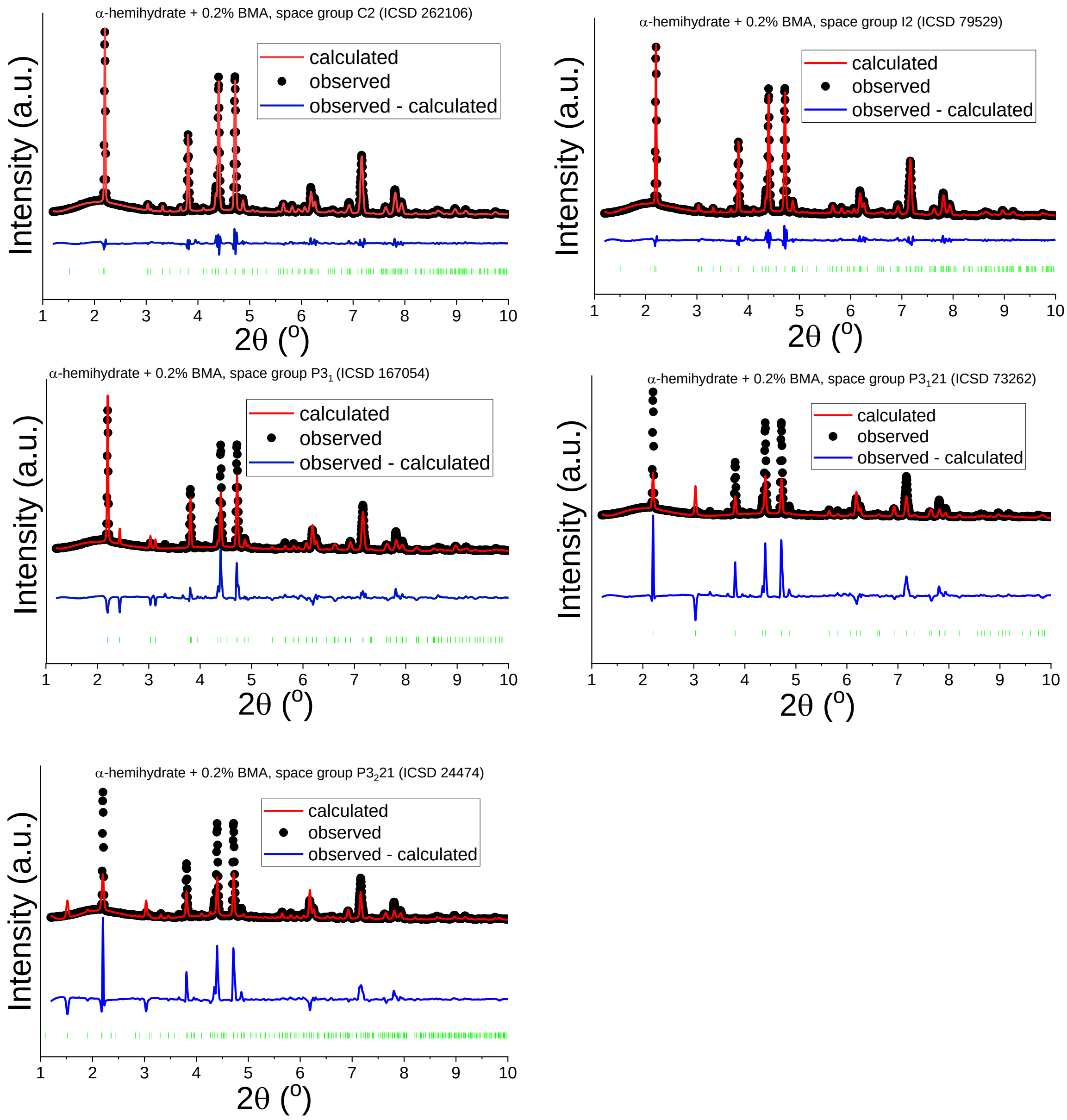


**Figure S11** The results of structural refinements with the different models reported in the ICSD for α-$CaSO_4 \cdot 0.5H_2O$ with 0.2 % w/w BMA.

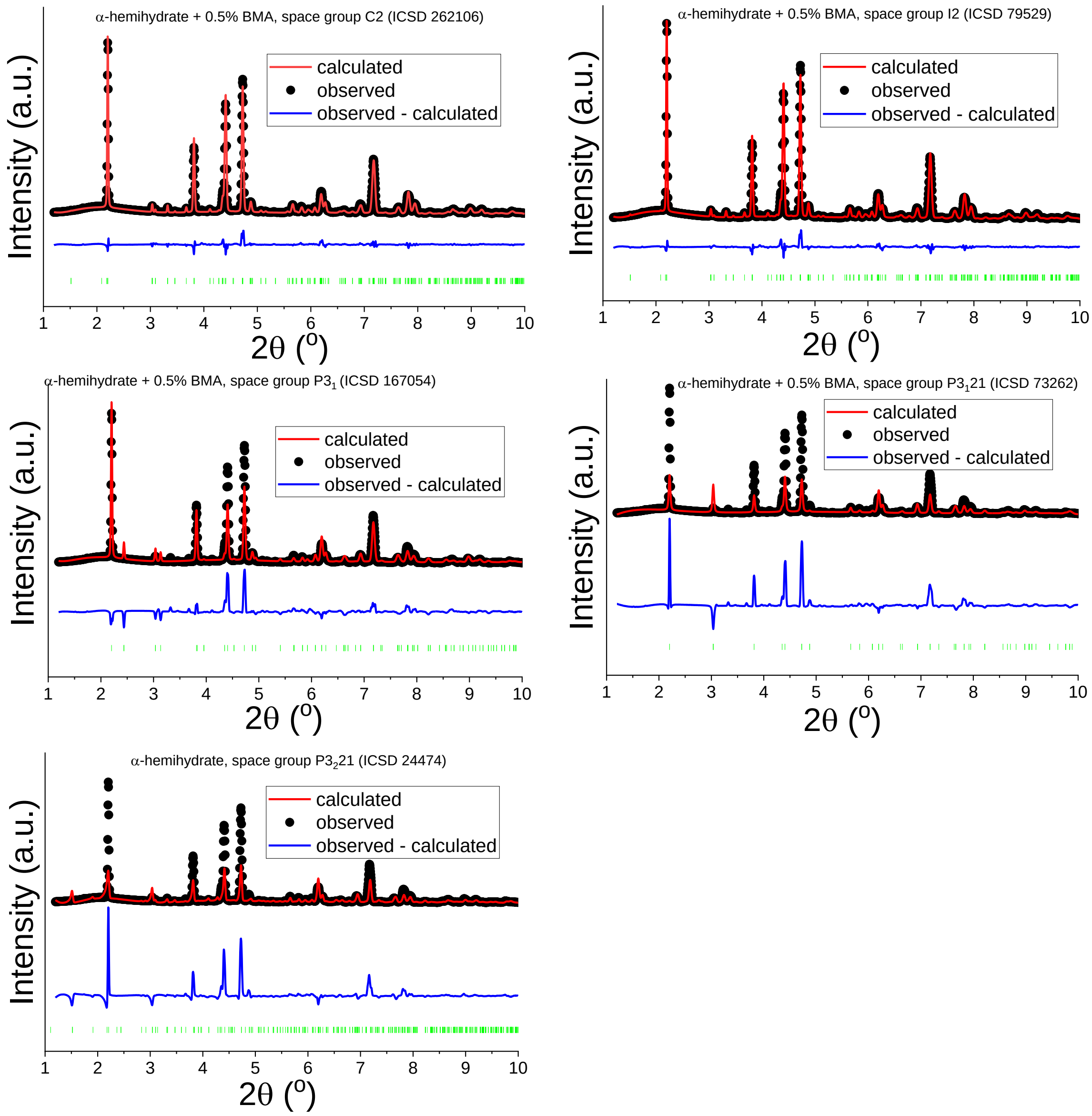


**Figure S12** The results of structural refinements with the different models reported in the ICSD for α-$CaSO_4 \cdot 0.5H_2O$ with 0.5 % w/w BMA.

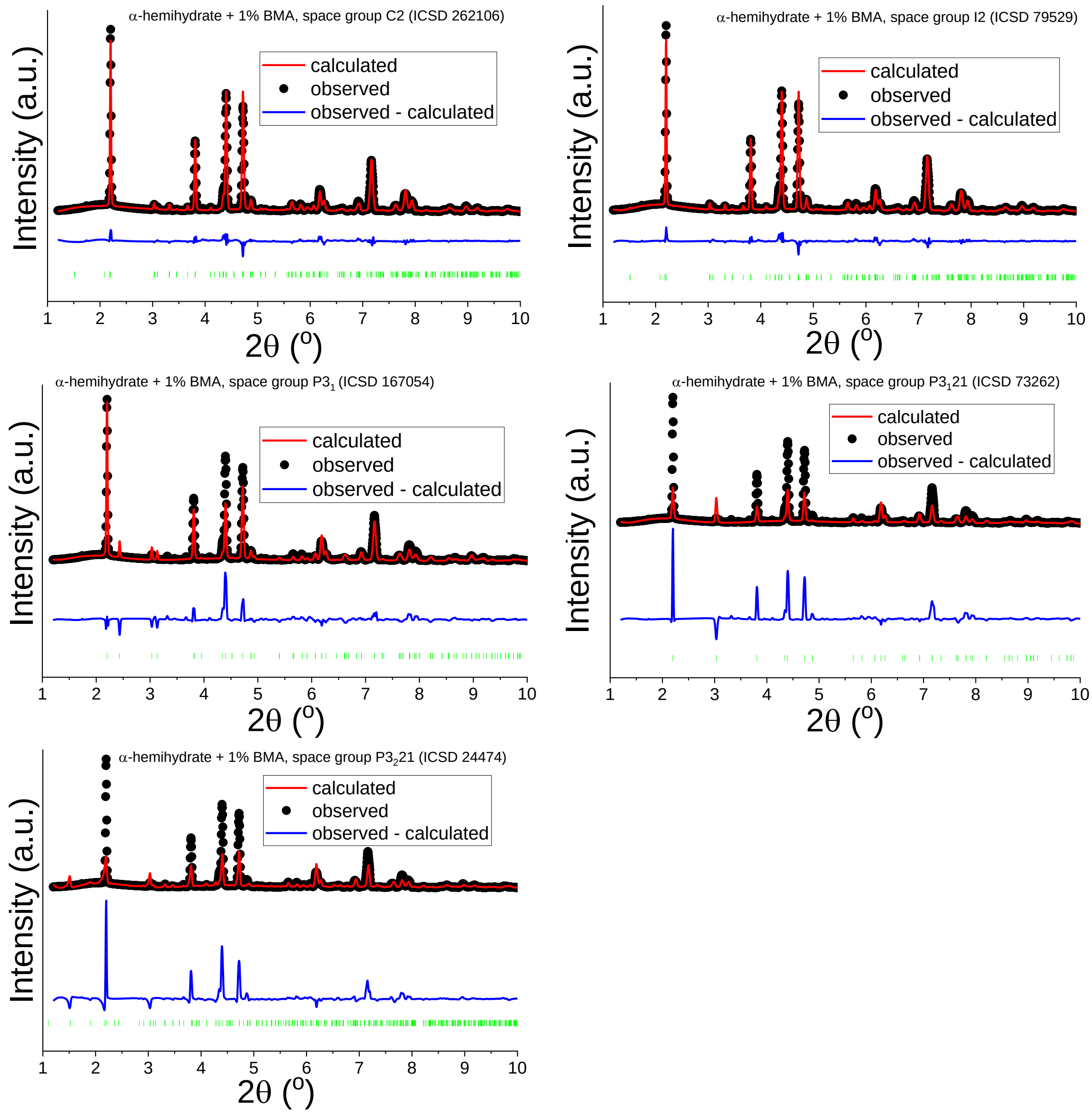


**Figure S13** The results of structural refinements with the different models reported in the ICSD for α-$CaSO_4 \cdot 0.5H_2O$ with 1 % w/w BMA.

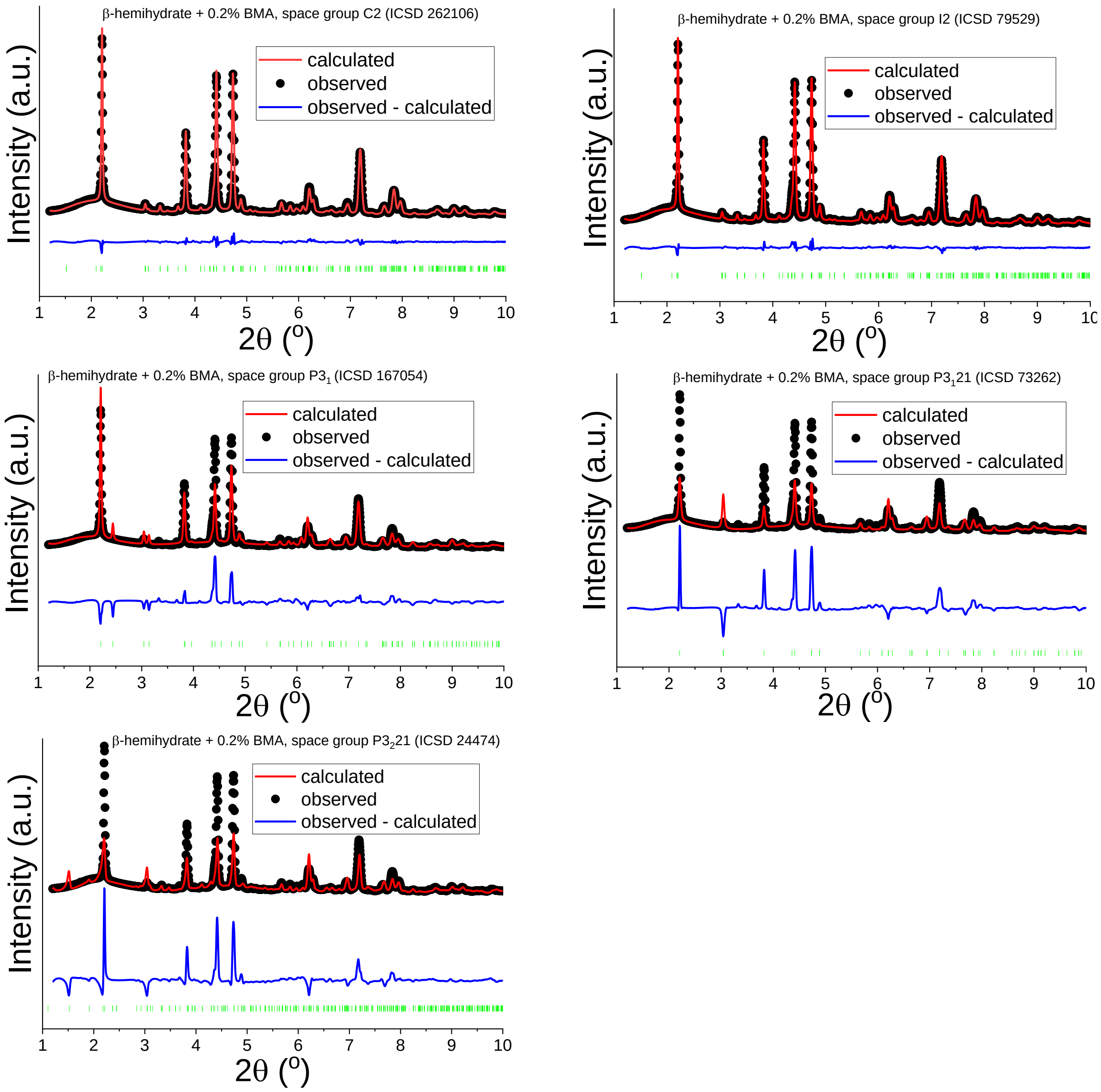


**Figure S14** The results of structural refinements with the different models reported in the ICSD for β-$CaSO_4 \cdot 0.5H_2O$ with 0.2 % w/w BMA.

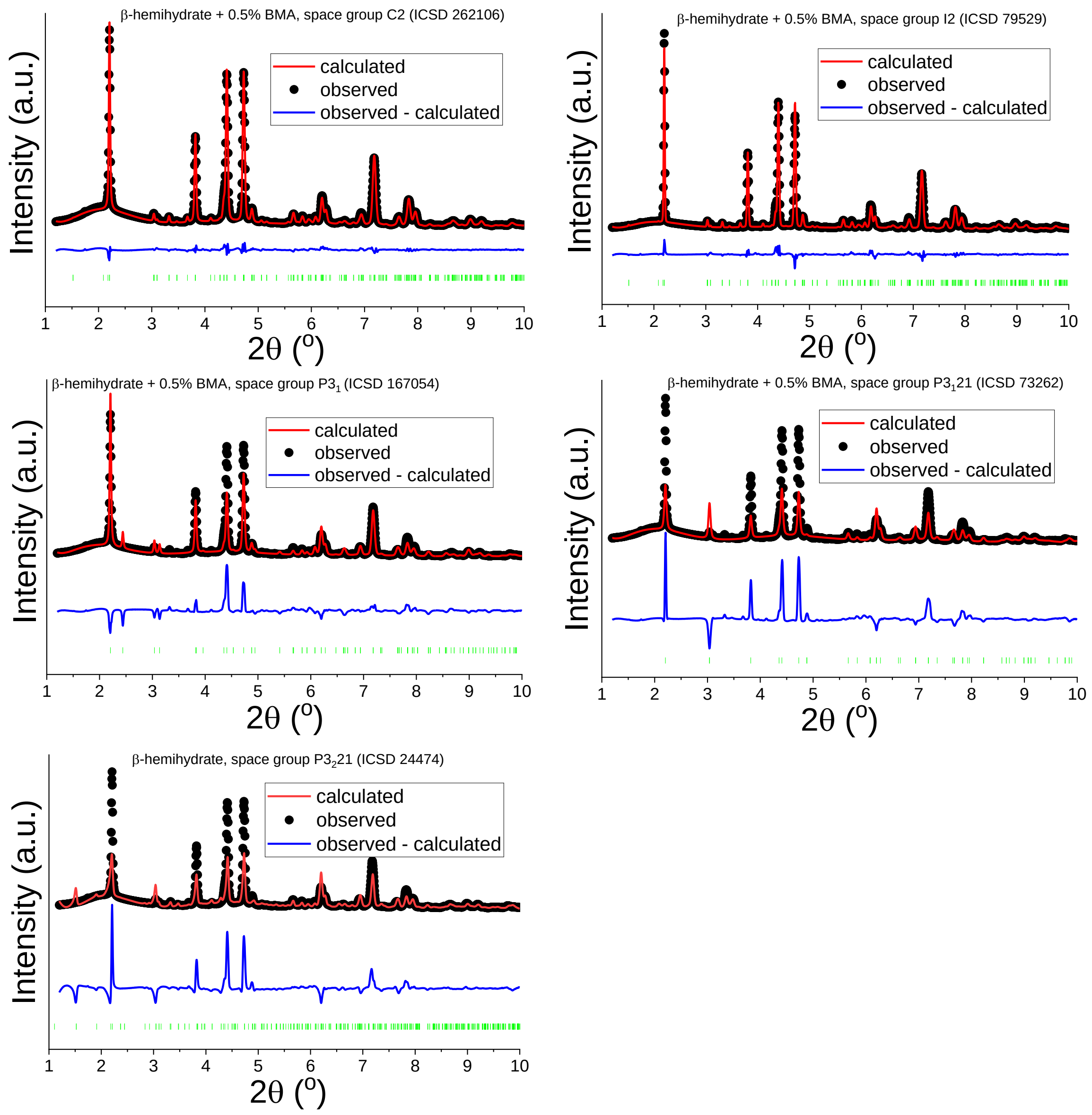


**Figure S15** The results of structural refinements with the different models reported in the ICSD for β-$CaSO_4 \cdot 0.5H_2O$ with 0.5 % w/w BMA.

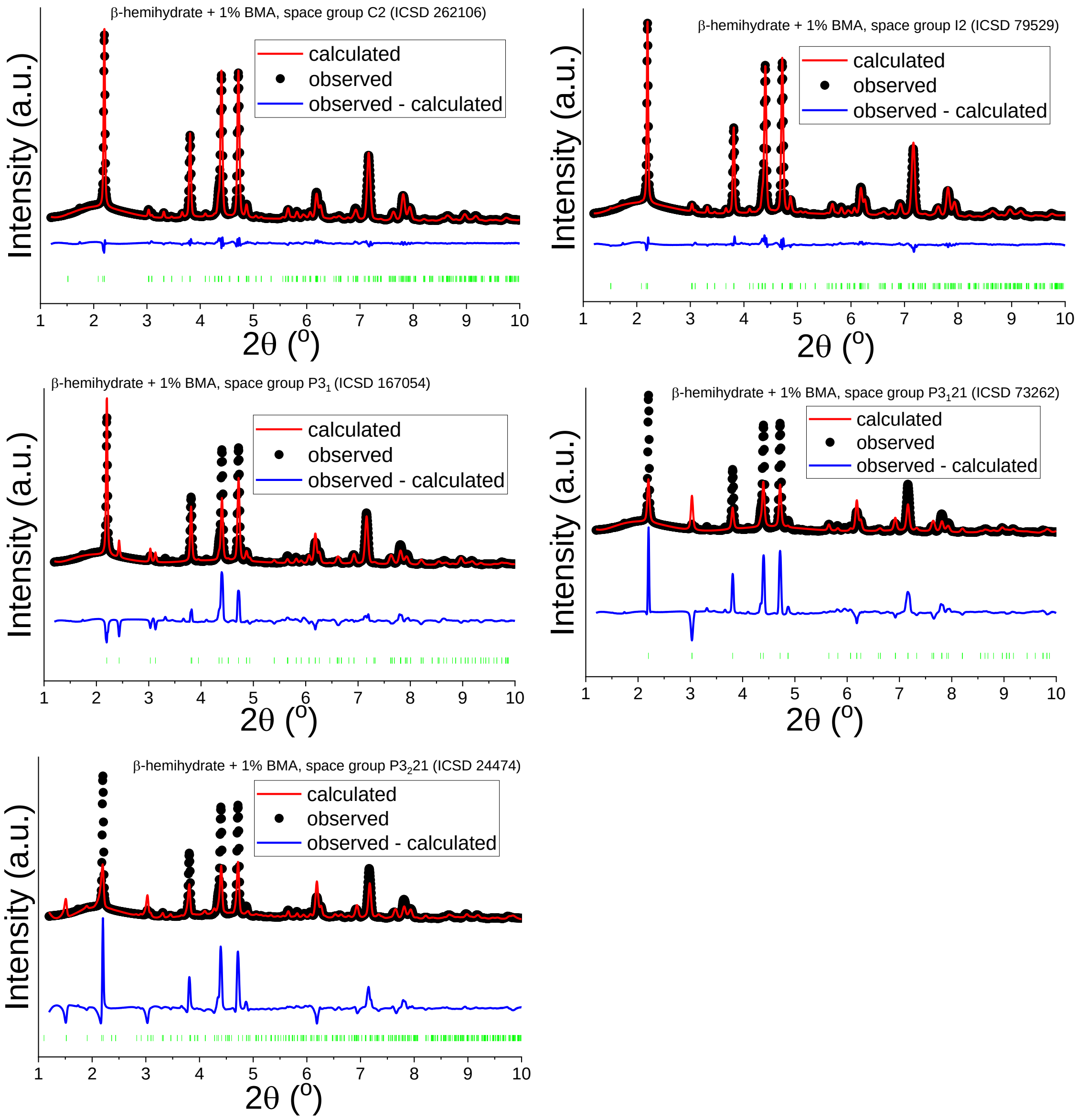


**Figure S16** The results of structural refinements with the different models reported in the ICSD for β-$CaSO_4$·$0.5H_2O$ with 1 % w/w BMA.

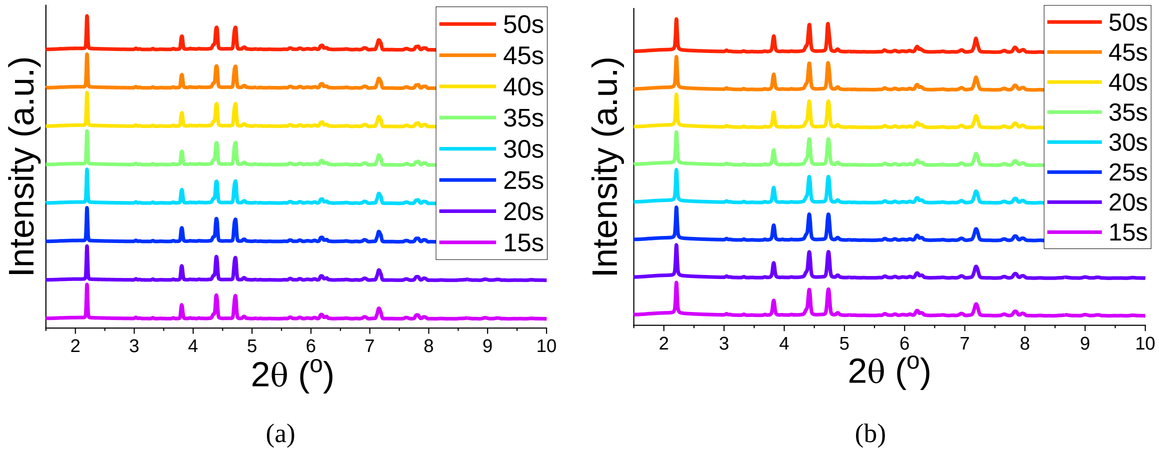


**Figure S17** The XRD patterns recorded at the synchrotron (a) for α-hemihydrate, water added after 25s; (b) for β-hemihydrate, water added after 20s.

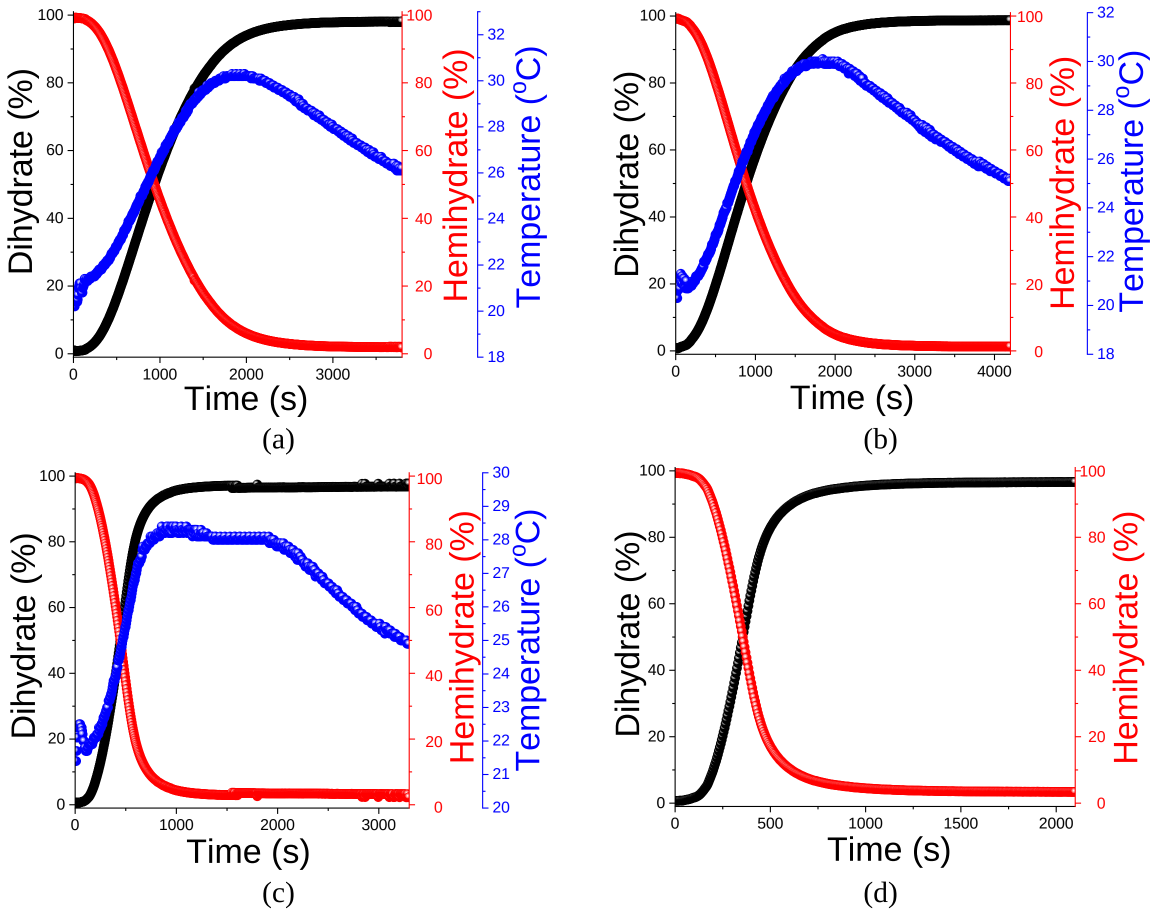


**Figure S18** In situ data showing the hydration of (a) α-$CaSO_4{\cdot}0.5H_2O$ + 0.2% BMA; (b) α-$CaSO_4{\cdot}0.5H_2O$ + 0.5% BMA; (c) β-$CaSO_4{\cdot}0.5H_2O$ + 0.2% BMA; (d) β-$CaSO_4{\cdot}0.5H_2O$ + 0.5% BMA (a fault with the probe meant that no accurate temperature data were recorded for his reaction). Phase fractions of the hemi- and dihydrate were determined by batch Rietveld refinements, and are plotted in percentage terms.

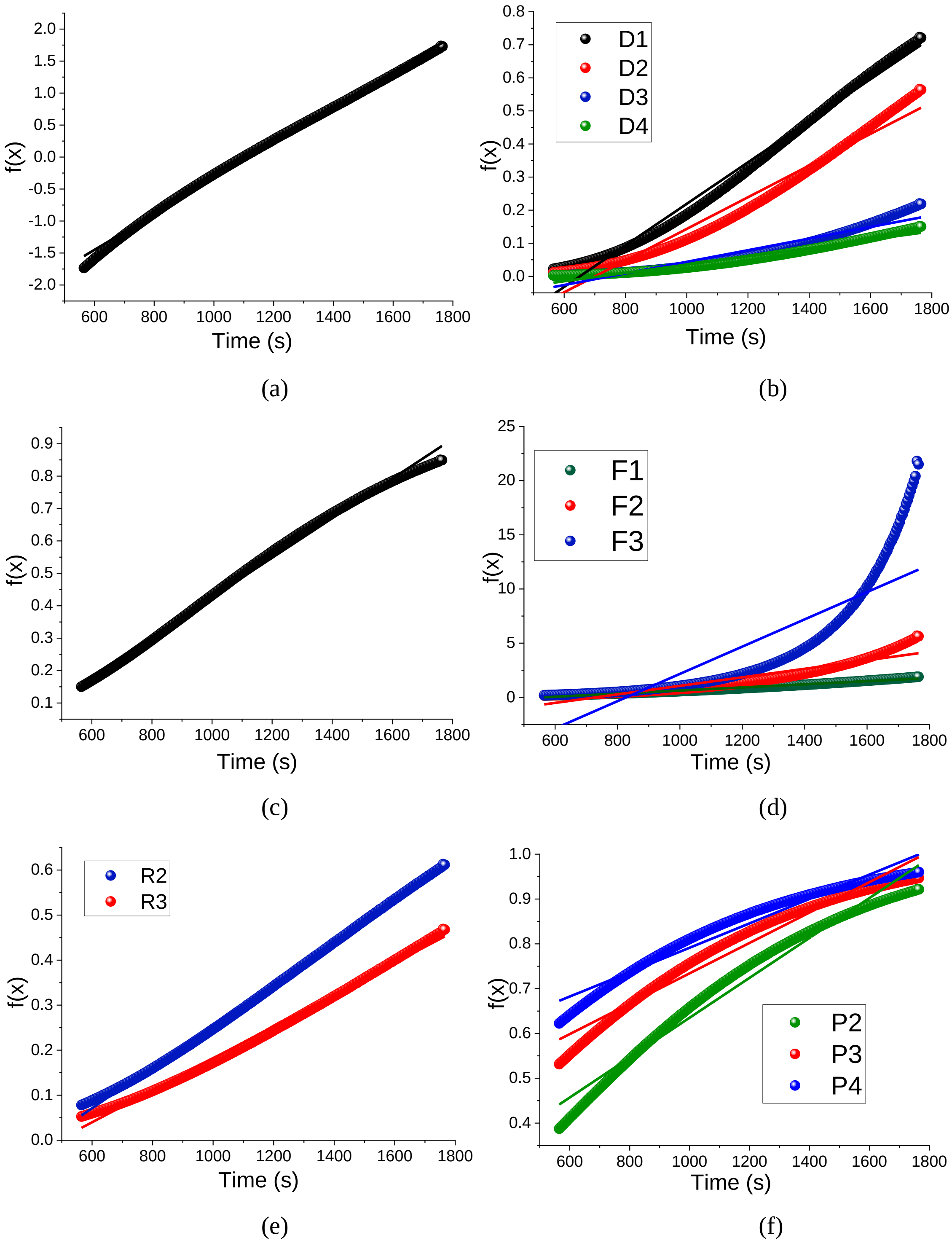


**Figure S19** Fits of selected kinetic models to the hydration of α-$CaSO_4 \cdot 0.5H_2O$, showing the (a) Prout-Tompkins; (b) diffusion; (c) zero-order; (d) first-, second- and third-order; (e) R2 contracting area and R3 contracting volume; and, (f) power law models.

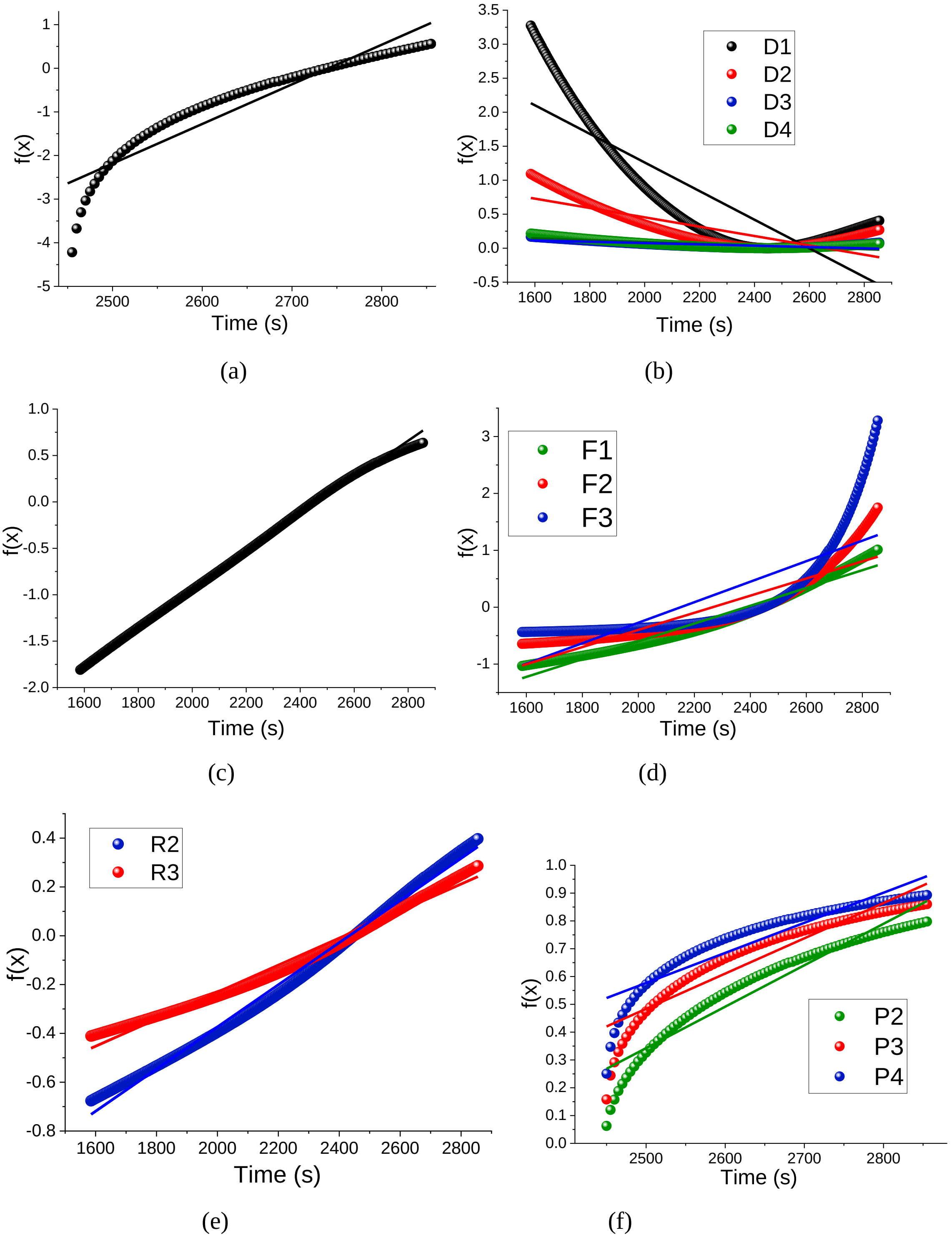


**Figure S20** Fits of selected kinetic models to the hydration of β-$CaSO_4 \cdot 0.5H_2O$, showing the (a) Prout-Tompkins; (b) diffusion; (c) zero-order; (d) first-, second- and third-order; (e) R2 contracting area and R3 contracting volume; and, (f) power law models.

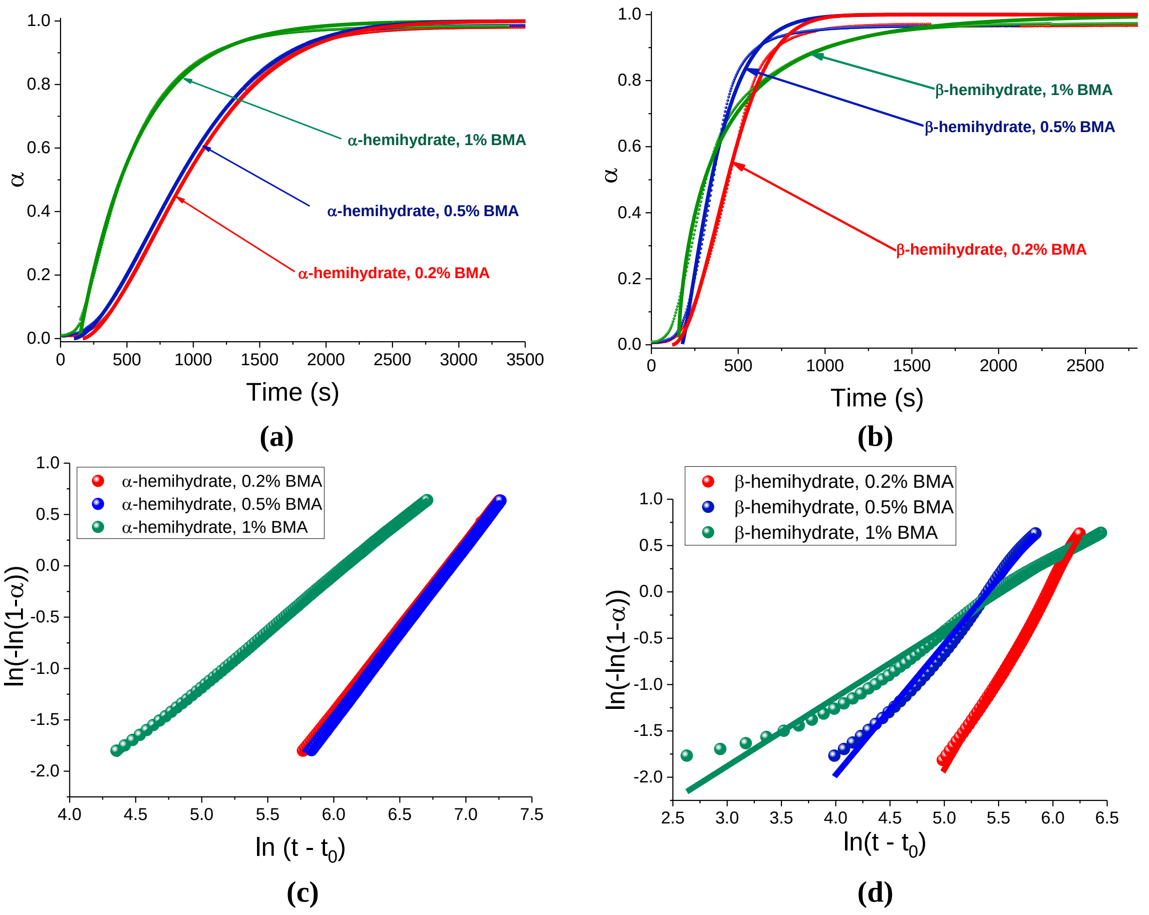


**Figure S21** Avrami-Erofe'ev fitting to accelerated hydration reactions, showing direct fits to the experimental data for (a) α-$CaSO_4{\cdot}0.5H_2O$ and (b) β-$CaSO_4{\cdot}0.5H_2O$ and Sharp-Hancock plots for (c) α-$CaSO_4{\cdot}0.5H_2O$ and (d) β-$CaSO_4{\cdot}0.5H_2O$.

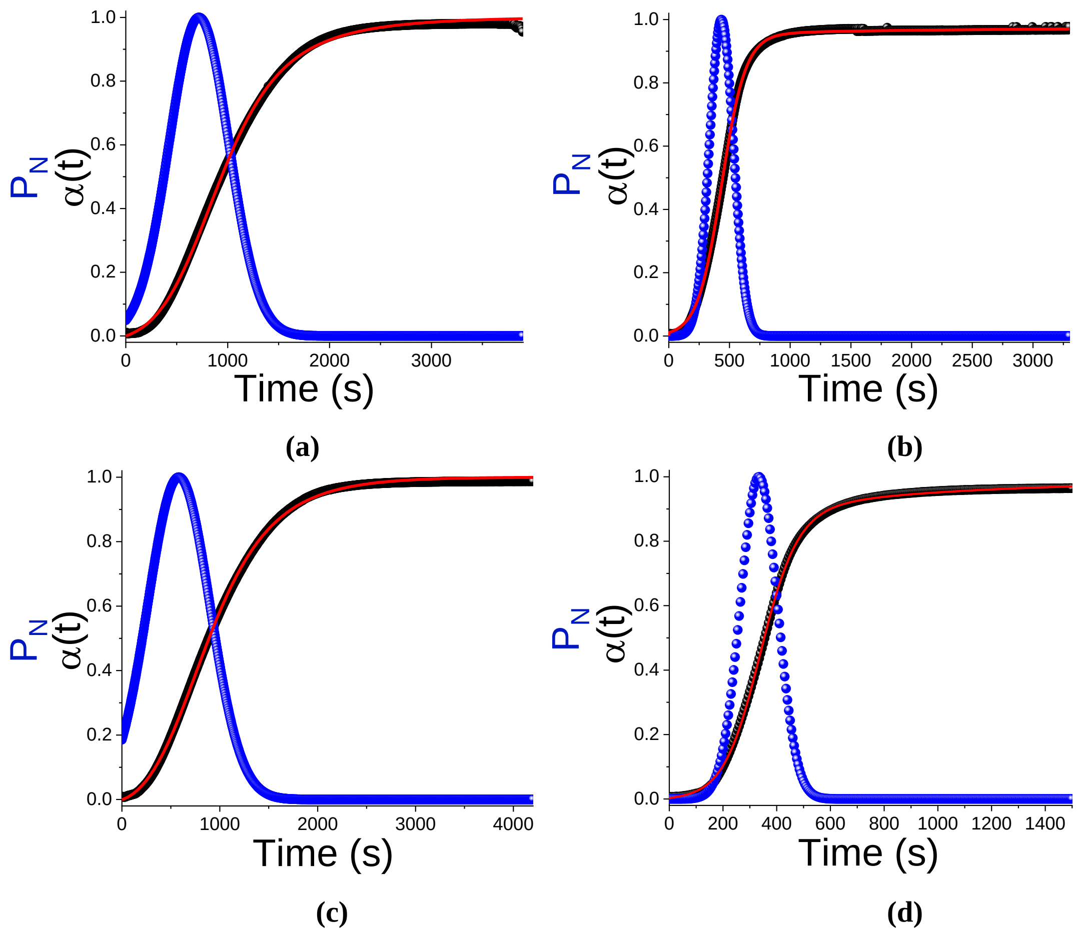


**Figure S22** Gualtieri fits for the hydration of **(a)** α-CaSO4·0.5H2O with 0.2 % w/w BMA; **(b)** β-CaSO4·0.5H2O with 0.2 % w/w BMA; **(c)** α-CaSO4·0.5H2O with 0.5 % w/w BMA; and, **(d)** β-CaSO4·0.5H2O with 0.5 % w/w BMA. Experimental data (■), corresponding Gualtieri fits (–) and the calculated rate of nucleation ($P_N$; ●) are depicted.

**Table S1** Refinement data for α- and β-$CaSO_4 \cdot 0.5H_2O$.

| Space group | α–hemihydrate | | | | | |
|---|---|---|---|---|---|---|
| | **a (Å)** | **b (Å)** | **c (Å)** | **α (º)** | **β (º)** | **γ (º)** |
| ***C2*** **ICSD 262106** | 17.379(2) | 6.904(1) | 11.992(1) | 90 | 133.421(7) | 90 |
| ***I2*** **ICSD 79529** | 11.942(1) | 6.869(1) | 12.569(0) | 90 | 90.221(2) | 90 |
| ***$P3_1$*** **ICSD 167054** | 6.917(2) | 6.914(1) | 12.623(2) | 90 | 90 | 120 |
| ***$P3_221$*** **ISCD 24474** | 13.83(2) | 13.800(2) | 12.596(4) | 90 | 90 | 120.1(1) |
| ***$P3_121$*** **ICSD 73262** | 6.910(1) | 6.918(9) | 6.313(3) | 90 | 90 | 120.0(1) |

| Space group | β-hemihydrate | | | | | |
|---|---|---|---|---|---|---|
| | **a (Å)** | **b (Å)** | **c (Å)** | **α (º)** | **β (º)** | **γ (º)** |
| ***C2*** **ICSD 262106** | 17.342(3) | 6.889(0) | 11.908(1) | 90 | 133.546(9) | 90 |
| ***I2*** **ICSD 79529** | 11.942(1) | 6.869(1) | 12.570(0) | 90 | 90.21(1) | 90 |
| ***$P3_1$*** **ICSD 167054** | 6.885(2) | 6.882(1) | 12.569(2) | 90 | 90 | 120 |
| ***$P3_221$*** **ISCD 24474** | 13.760(2) | 13.730(1) | 12.542(4) | 90 | 90 | 120.07(9) |
| ***$P3_121$*** **ICSD 73262** | 6.880(1) | 6.889(9) | 6.285(2) | 90 | 90 | 120.0(1) |

**Table S2** $R_{wp}$ factors for α- and β-$CaSO_4 \cdot 0.5H_2O$, with and without the presence of BMA.

| Sample | Space group C2 ICSD 262106 | I2 ICSD 79529 | $P3_1$ ICSD 167054 | $P3_221$ ICSD 24474 | $P3_121$ ICSD 73262 |
|---|---|---|---|---|---|
| α-$CaSO_4 \cdot 0.5H_2O$ | 4.737 | 7.660 | 20.146 | 35.400 | 36.640 |
| α-$CaSO_4 \cdot 0.5H_2O$ + 0.2 % BMA | 4.017 | 13.047 | 21.852 | 33.027 | 34.058 |
| α-$CaSO_4 \cdot 0.5H_2O$ + 0.5 % BMA | 4.626 | 4.735 | 19.196 | 34.608 | 31.382 |
| α-$CaSO_4 \cdot 0.5H_2O$ + 1 % BMA | 4.750 | 4.750 | 20.070 | 31.002 | 32.699 |
| β-$CaSO_4 \cdot 0.5H_2O$ | 3.868 | 4.581 | 25.444 | 39.949 | 40.311 |
| β-$CaSO_4 \cdot 0.5H_2O$ + 0.2 % BMA | 7.387 | 6.599 | 21.841 | 35.095 | 35.512 |
| β-$CaSO_4 \cdot 0.5H_2O$ + 0.5 % BMA | 7.712 | 7.211 | 25.534 | 39.387 | 39.712 |
| β-$CaSO_4 \cdot 0.5H_2O$ + 1 % BMA | 7.565 | 7.957 | 24.826 | 39.223 | 39.886 |

**Table S3** Refinement data for α- and β-dihydrate.

| Space group | α-dihydrate | | | | | |
|---|---|---|---|---|---|---|
| *C2/c* ICSD 15982 | **a (Å)** | **b (Å)** | **c (Å)** | **α (º)** | **β (º)** | **γ (º)** |
| | 6.260(2) | 15.154(7) | 5.656(2) | 90 | 114.13(4) | 90 |
| | **β-dihydrate** | | | | | |
| *C2/c* ICSD 15982 | **a (Å)** | **b (Å)** | **c (Å)** | **α (º)** | **β (º)** | **γ (º)** |
| | 6.2485(2) | 15.117(9) | 5.645(2) | 90 | 114.13(5) | 90 |

**Table S4** The full list of kinetic models explored.

| Kinetic model | Description | Function |
|---|---|---|
| AE | Avrami-Erofe'ev | $1 - e^{-k(t-t_0)^n}$ |
| B1 | Prout-Tompkins | $[-\ln(1 - \alpha)] + c^a$ |
| D1 | 1-D diffusion | $\alpha^2$ |
| D2 | 2-D diffusion | $((1 - \alpha)\ln(1 - \alpha)) + \alpha$ |
| D3 | 3-D diffusion-Jander | $(1 - (1 - \alpha)^{1/3})^2$ |
| D4 | Ginstling-Brounshtein | $1 - (2/3)\alpha - (1 - \alpha)^{2/3}$ |
| F0/R1 | Zero-order | $\alpha$ |
| F1 | First-order | $-\ln(1 - \alpha)$ |
| F2 | Second-order | $[1/(1 - \alpha)] - 1$ |
| F3 | Third-order | $(1/2)[(1 - \alpha)^{-2} - 1]$ |
| R2 | Contracting area | $1 - (1 - \alpha)^{1/2}$ |
| R3 | Contracting volume | $1 - (1 - \alpha)^{1/3}$ |
| P2 | Power law | $\alpha^{1/2}$ |
| P3 | Power law | $\alpha^{1/3}$ |
| P4 | Power law | $\alpha^{1/4}$ |

**Table S5** $R^2$ fitting values obtained when applying the full set of kinetic models for the hydration of α- and β-$CaSO_4 \cdot 0.5H_2O$.

| Kinetic model | α | α + 0.2% BMA | α + 0.5% BMA | α + 1% BMA | β | β + 0.2% BMA | β + 0.5% BMA | β + 1% BMA |
|---|---|---|---|---|---|---|---|---|
| A2 | 0.9999 | 0.9991 | 0.9793 | 0.9729 | 0.9688 | 0.9676 | 0.9579 | 0.9490 |
| A3 | 0.9976 | 0.9938 | 0.9397 | 0.9394 | 0.9297 | 0.9283 | 0.9203 | 0.9034 |
| A4 | 0.9944 | 0.9891 | 0.9072 | 0.9159 | 0.9016 | 0.9007 | 0.8957 | 0.8724 |
| B1 | 0.9964 | 0.9924 | 0.7907 | 0.8644 | 0.8284 | 0.8348 | 0.8478 | 0.8000 |
| D1 | 0.9863 | 0.9927 | 0.5064 | 0.3444 | 0.7161 | 0.6236 | 0.5387 | 0.2235 |
| D2 | 0.9653 | 0.9769 | 0.4430 | 0.2513 | 0.6720 | 0.5701 | 0.4599 | 0.1180 |
| D3 | 0.9241 | 0.9416 | 0.2939 | 0.0927 | 0.5409 | 0.6236 | 0.2809 | 0.0064 |
| D4 | 0.9530 | 0.9668 | 0.4043 | 0.2036 | 0.6409 | 0.5343 | 0.4131 | 0.0746 |
| F0/R1 | 0.9946 | 0.9894 | 0.9667 | 0.9218 | 0.9977 | 0.9896 | 0.9737 | 0.8403 |
| F1 | 0.9796 | 0.9876 | 0.9923 | 0.9986 | 0.9579 | 0.9772 | 0.9922 | 0.9764 |
| F2 | 0.8766 | 0.8969 | 0.8821 | 0.9364 | 0.8094 | 0.8449 | 0.8947 | 0.9861 |
| F3 | 0.7267 | 0.7565 | 0.7052 | 0.7922 | 0.6283 | 0.6700 | 0.7434 | 0.8931 |
| P2 | 0.9737 | 0.9624 | 0.9409 | 0.9287 | 0.9220 | 0.9204 | 0.9057 | 0.8906 |
| P3 | 0.9620 | 0.9488 | 0.8877 | 0.8863 | 0.8732 | 0.8721 | 0.8616 | 0.8364 |
| P4 | 0.9553 | 0.9411 | 0.8495 | 0.8599 | 0.8416 | 0.8416 | 0.8351 | 0.8025 |
| R2 | 0.9982 | 0.9996 | 0.996 | 0.9762 | 0.9926 | 0.9993 | 0.9976 | 0.9205 |
| R3 | 0.9946 | 0.9983 | 0.9988 | 0.9877 | 0.9845 | 0.9956 | 0.9993 | 0.9425 |
| Gualtieri | 0.9994 | 0.9992 | 0.9995 | 0.9989 | 0.9992 | 0.9996 | 0.9998 | 0.9991 |
